# Dual pathways of tRNA hydroxylation ensure efficient translation by expanding decoding capability

**DOI:** 10.1038/s41467-019-10750-8

**Published:** 2019-06-28

**Authors:** Yusuke Sakai, Satoshi Kimura, Tsutomu Suzuki

**Affiliations:** Department of Chemistry and Biotechnology, Graduate School of Engineering, University of Tokyo, 7-3-1 Hongo, Bunkyo-ku, Tokyo 113-8656 Japan

**Keywords:** Bacterial genetics, Bacterial genomics, RNA modification, tRNAs

## Abstract

In bacterial tRNAs, 5-carboxymethoxyuridine (cmo^5^U) and its derivatives at the first position of the anticodon facilitate non-Watson–Crick base pairing with guanosine and pyrimidines at the third positions of codons, thereby expanding decoding capabilities. However, their biogenesis and physiological roles remained to be investigated. Using reverse genetics and comparative genomics, we identify two factors responsible for 5-hydroxyuridine (ho^5^U) formation, which is the first step of the cmo^5^U synthesis: TrhP (formerly known as YegQ), a peptidase U32 family protein, is involved in prephenate-dependent ho^5^U formation; and TrhO (formerly known as YceA), a rhodanese family protein, catalyzes oxygen-dependent ho^5^U formation and bypasses cmo^5^U biogenesis in a subset of tRNAs under aerobic conditions. *E. coli* strains lacking both *trhP* and *trhO* exhibit a temperature-sensitive phenotype, and decode codons ending in G (GCG and UCG) less efficiently than the wild-type strain. These findings confirm that tRNA hydroxylation ensures efficient decoding during protein synthesis.

## Abstract (web summary)

5-carboxymethoxyuridine (cmo5U) is one of the RNA modifications found in bacterial tRNA anticodons. Here the authors show that the first step of cmo5U biosynthesis from uridine is mediated by either one of two parallel factors, TrhP or TrhO, and that cmo5U modification is required for efficient translation.

## Introduction

RNA modifications confer chemical diversity on simple RNA molecules, expanding their functional repertoires. To date, more than 140 species of RNA modifications have been identified in RNA molecules from all domains of life^1^.

In protein synthesis, tRNA serves as an adapter molecule to connect codons on mRNA with the corresponding amino acids. After they are transcribed, tRNAs undergo chemical modifications mediated by site-specific tRNA-modifying enzymes. These modifications play critical roles in stabilizing tRNA tertiary structure and fine-tuning the decoding process^2,3^. A wide variety of modifications are present at the first (wobble) position of the anticodon in tRNA (position 34). The wobble modification modulates codon recognition, thereby promoting accurate decoding during protein synthesis^3,4^.

In the original wobble rule^5^, guanosine at the wobble position (G34) base-pairs with U and C at the third letter of the codon, likewise, uridine at the wobble position (U34) base-pairs with A and G at the third letter of the codon. In *Mycoplasma* species and mitochondria^6–8^, however, U34 recognizes any of the four bases in a family box through a mechanism called four-way wobbling^3^. To decode two codon sets ending in purine (NNR), U34 is frequently modified to 5-methyl-(2-thio)uridine derivatives [xm^5^(s^2^)U]: 5-carboxymethylaminomethyl-(2-thio)uridine [cmnm^5^(s^2^)U] and 5-methylaminomethyl-(2-thio)uridine [mnm^5^(s^2^)U] in bacterial tRNAs, 5-methoxycarbonylmethyl-(2-thio)uridine [mcm^5^(s^2^)U] and its derivatives in eukaryotic cytoplasmic tRNAs, and 5-taurinomethyl-(2-thio)uridine [τm^5^(s^2^)U] in mitochondrial tRNAs ^3^. These modifications prevent tRNAs from misreading near-cognate codons ending in pyrimidines (NNY)^9^. In addition, xm^5^(s^2^)U34 plays a critical role in stabilizing U-G wobble pairing at the A-site of the ribosome^10,11^.

**Fig. 1:**
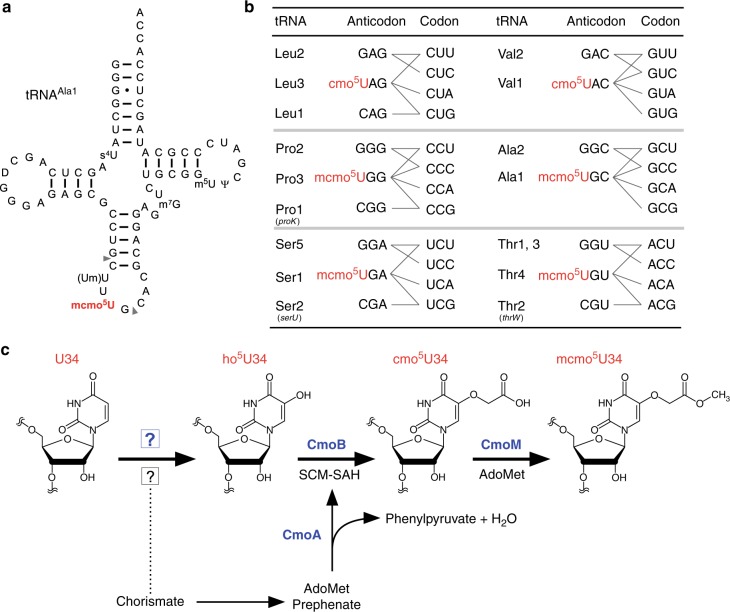
Decoding capacity of tRNAs is expanded by (m)cmo^5^U. **a** Secondary structure of *E. coli* tRNA^Ala1^ with post-transcriptional modifications: 4-thiouridine (s^4^U), dihydrouridine (D), 2’-*O*-methyluridine (Um), 5-methoxycarbonylmethoxyuridine (mcmo^5^U), 7-methylguanosine (m^7^G), 5-methyluridine (m^5^U), and pseudouridine (Ψ). A pair of gray triangles indicate the RNase T_1_ cleavage positions for analysis of RNA fragments containing the wobble position. **b** Anticodon–codon pairing in six codon boxes decoded by tRNAs bearing cmo^5^U and mcmo^5^U. Each of the three tRNA genes in parenthesis was individually deleted. In the family boxes (four codons specify a single amino acid), many codons are redundantly decoded by two or three isoacceptors (tRNAs charging with the same amino acid). For example, CUU codon is decoded by tRNA^Leu2^ with GAG anticodon as well as tRNA^Leu3^ with cmo^5^UAG anticodon. **c** Scheme of mcmo^5^U biosynthesis in *E. coli*. First, U34 is hydroxylated by reactions involving unknown enzymes and substrates to form ho^5^U34 in tRNAs responsible for decoding NYN codons. This reaction requires the chorismate biogenesis pathway. CmoA employs prephenate and AdoMet to generate SCM-SAH with the release of phenylpyruvate and water. Then, ho^5^U34 is carboxymethylated by CmoB using SCM-SAH. Finally, tRNAs responsible for decoding NCN codons are further methylated to generate mcmo^5^U by CmoM in the presence of AdoMet

In contrast to xm^5^(s^2^)U-type modifications, 5-hydroxyuridine derivatives (xo^5^U) are present at the wobble positions of tRNAs responsible for NYN family boxes, and serve to expand decoding capacity in most bacterial species (Fig. 1a, b). To date, four species of xo^5^U modifications have been reported. 5-carboxymethoxyuridine (cmo^5^U, also called uridine-5-oxy acetic acid) and 5-methoxycarbonylmethoxyuridine (mcmo^5^U, also called uridine-5-oxy acetic acid methyl ester) are found in Gram-negative bacteria, including *Escherichia coli* and *Salmonella enterica*^2,12^. 5-methoxycarbonylmethoxy-2’*O*-methyluridine (mcmo^5^Um) was recently detected in *E. coli* tRNA^Ser^ as a minor modification^13^. In addition, 5-methoxyuridine (mo^5^U) is present in tRNA^Thr^ from Gram-positive bacteria, including *Bacillus subtilis*^14^. Using mass spectrometry, we revealed that cmo^5^U34 is present as a major wobble modification in tRNA^Leu3^ and tRNA^Val1^, whereas mcmo^5^U34 is primarily present in tRNA^Ala1^, tRNA^Ser1^, tRNA^Pro3^, and tRNA^Thr4^ in *E. coli*^13^ (Fig. 1b). The xo^5^U-type modifications facilitate non-Watson–Crick base pairing with guanosine and pyrimidines at the third positions of codons^15–18^ (Fig. 1b), thereby contributing to expansion of decoding capability. Moreover, the terminal methylation of mcmo^5^U contributes to decoding ability, at least in tRNA^Ala1^ ^13^.

A structural study of cmo^5^U nucleoside in solution revealed that cmo^5^U adopts the C2’-*endo* ribose pucker conformation, providing mechanistic insight into base pairing with pyrimidines^9^. The crystal structures of codon–anticodon interactions at the ribosomal A-site revealed that the O5 atom of cmo^5^U is involved in an intramolecular hydrogen bond that pre-structures the anticodon loop, enabling cmo^5^U to pair with pyrimidines at the third position of the codon^19^. In addition, cmo^5^U pairs with G in standard Watson–Crick geometry, rather than classical U-G wobble geometry, indicating that keto-to-enol tautomeric conversion of the uracil base is involved in this base pairing interaction^19^.

Multistep reactions are involved in the biosynthetic pathway of cmo^5^U and mcmo^5^U in bacterial tRNAs (Fig. 1c). Three enzymes, CmoA, CmoB, and CmoM, are involved in the second to last step in the pathway. Initially, U34 is hydroxylated to form 5-hydroxyuridine (ho^5^U) in a reaction catalyzed by unidentified factors. Chorismate, an end product of the shikimate pathway, is involved in this step, but the source of the oxygen atom remains unknown^20^. On the other hand, the subsequent steps have been elucidated. In the second step, a unique carboxymethyl donor, *S*-carboxymethyl-*S*-adenosyl-l-homocysteine (SCM-SAH, also called Cx-SAM) is synthesized from AdoMet and prephenate in a reaction catalyzed by CmoA^21,22^. CmoB then transfers the carboxymethyl group of SCM-SAH to ho^5^U34 to form cmo^5^U34 on tRNA^21^. In four tRNA species (for Ser1, Pro3, Thr4, and Ala1), cmo^5^U is further methylated by CmoM to form mcmo^5^U34^13^. The terminal methylation of mcmo^5^U in tRNA^Pro3^ dynamically alters its modification frequency in a growth phase-dependent manner^13^.

Hydroxylation of RNA molecules is a major post-transcriptional modification that plays roles in multiple biological contexts. In addition to xo^5^U modification of bacterial tRNAs, RNA hydroxylation is also responsible for the biogenesis and function of eukaryotic RNA modifications. The JmjC domain-containing protein TYW5 is responsible for biogenesis of hydroxywybutosine (OHyW) at position 37 of mammalian tRNA^Phe^^23^. ALKBH1 catalyzes tRNA hydroxylation to form 5-hydroxymethyl-2’*O*-methylcytidine (hm^5^Cm) and 5-formyl-2’*O*-methylcytidine (f^5^Cm) in cytoplasmic tRNA^Leu^, as well as 5-formylcytidine (f^5^C) in mammalian mitochondrial tRNA^Met^^24^. ALKBH1-knockout cells exhibit respiratory defects, indicating that ALKBH1 is required for efficient mitochondrial activity. Recently, we discovered hydroxy-*N*^6^-threonylcarbamoyladenosine (ht^6^A) in echinoderm mitochondrial tRNA;^25^ this modification alters the genetic code in echinoderm mitochondria. *N*^6^-methyladenosine (m^6^A) and 1-methyladenosine (m^1^A) are demethylated by ALKBH family proteins via hydroxymethyl formation^26,27^. The Tet protein is responsible for forming 5-hydroxymethylcytidine (hm^5^C) in *Drosophila* mRNAs^28^. All of the hydroxylases, including the JmjC, ALKBH, and Tet families, are Fe(II)- and 2-oxoglutarate (2-OG)-dependent oxygenases that use a molecular oxygen as a substrate for hydroxylation^29^. Recently, we identified RlhA as a factor responsible RNA hydroxylation to form 5-hydroxycytidine (ho^5^C) at position 2501 in *E. coli* 23S rRNA^30^. RlhA does not have any characteristic motifs conserved among the known RNA oxygenases, but it does belong to a family of proteins bearing the peptidase U32 motif. This finding encouraged us to explore other peptidase U32-containing proteins responsible for hydroxylation of biological molecules.

To search for genes responsible for RNA modifications, we developed a method called ribonucleome analysis to screen knockout strains for uncharacterized genes by liquid chromatography–mass spectrometry (LC/MS)^31^. If a target RNA modification is absent in a certain knockout strain, we can identify the gene dedicated to biogenesis of the target RNA modification via reverse genetics. Using this approach, we have discovered dozens of genes responsible for RNA modifications in tRNAs^13,23,32–39^ and rRNAs^30,40,41^. In this study, we apply ribonucleome analysis in conjunction with comparative genomics to identify two genes, *trhP* and *trhO*, which are responsible for formation of ho^5^U at the initial step of xo^5^U biogenesis at the wobble position of bacterial tRNAs. TrhP, a peptidase U32 family protein, is involved in prephenate-dependent ho^5^U34 formation. TrhO, a rhodanese family protein, catalyzes oxygen-dependent ho^5^U34 formation under aerobic conditions. These two pathways play redundant roles in ho^5^U34 formation. Double knockout of both enzymes causes a temperature-sensitive phenotype and decreases the efficiency with which codons ending in G (GCG and UCG) were decoded, indicating that ho^5^U34 formation ensures efficient decoding during translation.

## Results

### *E. coli yegQ* is involved in tRNA hydroxylation

**Fig. 2:**
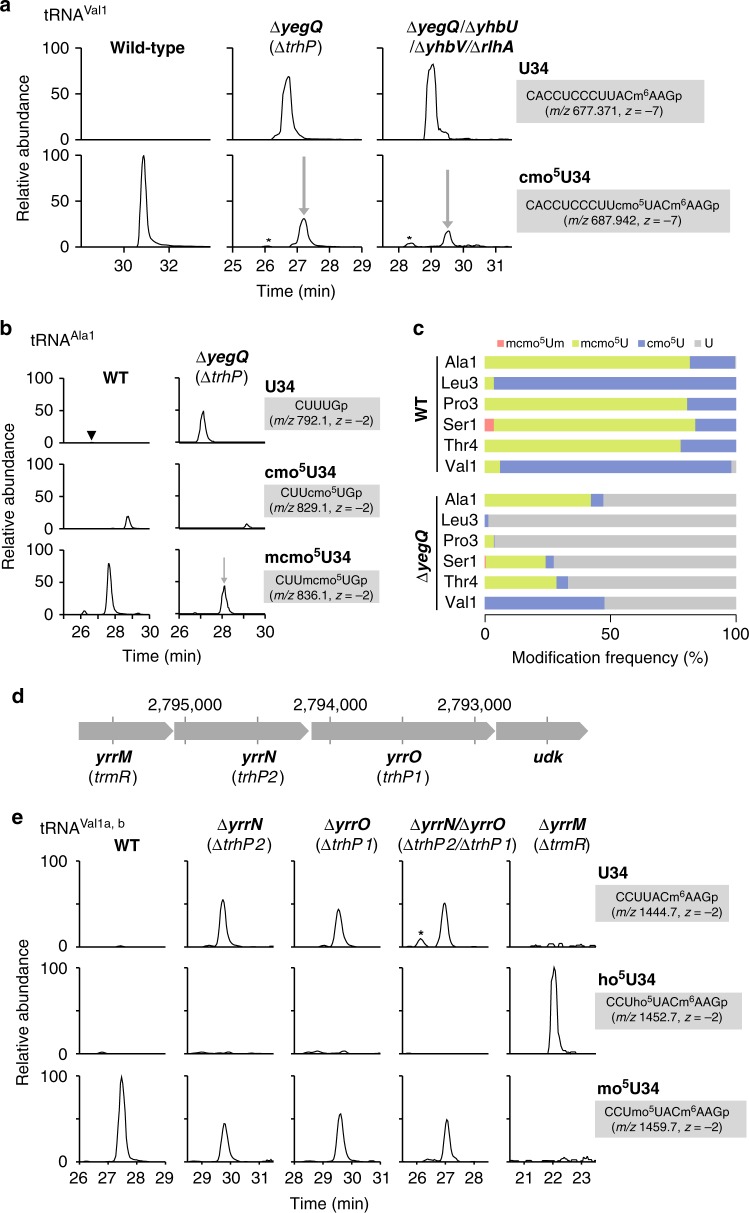
Identification of *trhP* responsible for tRNA hydroxylation. **a** Mass-spectrometric shotgun analysis of total tRNAs in *E. coli* strains. Extracted ion chromatograms (XICs) show multiply charged negative ions of the anticodon-containing fragments of tRNA^Val1^ with U34 (upper panels) and cmo^5^U34 (lower) in total tRNAs from wild-type (left), ∆*yegQ* (center), and ∆*yegQ*/∆*yhbU*/∆*yhbV*/∆*rlhA* strains (right). Sequence, *m/z* value, and charge state of each fragment are shown on the right. Asterisks indicate nonspecific peaks with the same *m/z* values. **b** Mass-spectrometric analysis of the wobble modification in *E. coli* tRNA^Ala1^ isolated from WT (left panels) and ∆*yegQ* (right) strains. XICs show anticodon-containing fragments of tRNA^Ala1^ with U34 (top panels), cmo^5^U34 (middle panels), and mcmo^5^U34 (bottom panels). The cleavage sites of RNase T_1_ are shown in Fig. 1a. It is assumed that cmo^5^U detected in this tRNA was generated by artificial hydrolysis of mcmo^5^U during tRNA isolation^13^. The black arrowhead indicates a small peak corresponding to the U34-containing fragment detected in the WT. **c** Modification frequencies of cmo^5^U derivatives at the wobble position of six tRNA species isolated from WT and ∆*yegQ* strains. Relative compositions of each modification were calculated from the peak area ratio of mass chromatograms of RNase T_1_ digest fragments containing mcmo^5^Um (red), mcmo^5^U (green), cmo^5^U (blue), or U (gray) at the wobble position (Supplementary Fig. 2)^13^. Source data are provided as a Source Data file. **d** Gene organization of a *B. subtilis* operon containing *yrrM*, *yrrN*, and *yrrO*. Genomic positions in *B. subtilis* are indicated. The new name for each gene is shown in parenthesis. **e** Mass-spectrometric shotgun analysis of total tRNAs in *B. subtilis* strains. XICs are shown of doubly charged negative ions of the anticodon-containing fragments of tRNA^Val1a,b^ with U34 (top panels), ho^5^U34 (middle panels), and mo^5^U34 (bottom panels) in total tRNA from wild-type (leftmost panels), ∆*yrrN* (left panels), ∆*yrrO* (middle panels), ∆*yrrN*/∆*yrrO* (right panels), and ∆*yrrM* (rightmost panels) strains

We recently reported that RlhA is responsible for ho^5^C formation at position 2501 in *E. coli* 23S rRNA^30^. RlhA belongs to a family of proteins that contain the peptidase U32 motif. This finding prompted us to speculate that other paralogs of peptidase U32-containing proteins are involved in ho^5^U34 formation in tRNAs. The *E. coli* genome contains four paralogs of peptidase U32-containing proteins: *rlhA*, *yegQ*, *yhbU*, and *yhbV* (Supplementary Fig. 1, Supplementary Data 1). We extracted total RNA from each of the respective knockout strains, digested with RNase T_1_, and subjected the digests to capillary LC-nano-ESI-mass spectrometry (RNA-MS) to detect tRNA fragments containing cmo^5^U (shotgun analysis). RNA fragments were detected as multiply charged negative ions (Supplementary Table 1). We clearly detected an anticodon-containing RNA fragment of tRNA^Val1^ from total RNA of wild-type (WT) *E. coli* cells (Fig. 2a). As reported previously^13^, cmo^5^U34 was present as a major wobble modification in this tRNA, whereas little unmodified fragment (U34) was detected. In a knockout strain lacking *yegQ* (∆*yegQ*), which encodes a peptidase U32-containing protein, cmo^5^U34 frequency decreased markedly, to about 30% of the WT level, and a corresponding fragment containing unmodified U34 appeared, indicating that *yegQ* is partially responsible for the initial step of cmo^5^U34 formation. We reasoned that other paralogs of peptidase U32-containing proteins might be involved in this process. Accordingly, we constructed a quadruple mutant strain, ∆*yegQ*/∆*yhbU*/∆*yhbV*/∆*rlhA*, lacking all paralogs of peptidase U32-containing proteins, and analyzed the RNA fragment of tRNA^Val1^. However, the cmo^5^U34-containing fragment persisted in this strain (Fig. 2a), clearly indicating that another hydroxylation pathway plays a redundant role in the formation of ho^5^U34 in tRNAs.

To analyze the modification status of each tRNA species in the ∆*yegQ* strain, we used the reciprocal circulating chromatography (RCC) method^42^ to isolate six tRNA species (tRNA^Ala1^, tRNA^Leu3^, tRNA^Pro3^, tRNA^Ser1^, tRNA^Thr4^, and tRNA^Val1^) bearing a cmo^5^U34 or mcmo^5^U34 modification from both WT and ∆*yegQ* strains. Each tRNA was digested by RNase T_1_ and subjected to RNA-MS to analyze the anticodon-containing fragments (Fig. 2b, Supplementary Fig. 2, Supplementary Table 1). Hypomodified fragments were further sequenced by collision-induced dissociation (CID) analysis (Supplementary Fig. 3). In tRNA^Ala1^, mcmo^5^U34 was present as a major wobble modification in the WT, but its levels were reduced to about 50% (concomitant with appearance of the unmodified fragment) in ∆*yegQ* (Fig. 2b). Similarly, mcmo^5^U34 levels were reduced in tRNA^Ser1^ and tRNA^Thr4^ from ∆*yegQ* (Fig. 2c, Supplementary Fig. 2). The level of cmo^5^U34, a major modification in WT tRNA^Val1^, was also reduced about 50% in ∆*yegQ* (Fig. 2c, Supplementary Fig. 2). By contrast, the levels of cmo^5^U34 of tRNA^Leu3^ and mcmo^5^U34 of tRNA^Pro3^ dropped sharply, to less than 5% of WT levels, in the ∆*yegQ* strain (Fig. 2c, Supplementary Fig. 2). These results indicate that *yegQ* is involved differently in ho^5^U34 formation in each tRNA. In particular, tRNA^Leu3^ and tRNA^Pro3^ are major targets of YegQ. Accordingly, we renamed *yegQ* as *trhP* (tRNA hydroxylation P).

### Biogenesis of mo^5^U34 in *Bacillus subtilis*

Instead of cmo^5^U34 or mcmo^5^U34, *B. subtilis* uses mo^5^U34 as an xo^5^U-type wobble modification. Upon depletion of intracellular AdoMet, mo^5^U is replaced by ho^5^U^43^, indicating that ho^5^U is the precursor and AdoMet is the methyl donor for mo^5^U formation. We identified two orthologs of *trhP* in *B. subtilis*, *yrrN* and *yrrO* (Supplementary Fig. 1, Supplementary Data 1), both of which are encoded tandemly in the same operon (Fig. 2d). YrrO has a peptidase U32 motif and a characteristic C-terminal motif also found in *E. coli* TrhP, whereas YrrN only has a peptidase U32 motif.

We investigated whether these two genes are actually involved in mo^5^U34 formation by shotgun analysis of total RNAs extracted from *B. subtilis* ∆*yrrN* and ∆*yrrO* strains. Similar to our observation in the *E. coli* ∆*trhP* strain (Fig. 2a), mo^5^U34 frequency in tRNA^Val1^ decreased, and an RNA fragment containing unmodified U34 appeared, in both ∆*yrrN* and ∆*yrrO* strains (Fig. 2e), indicating that both *yrrN* and *yrrO* were necessary for hydroxylation of U34 to form ho^5^U34 in *B. subtilis*. Accordingly, we renamed *yrrO* and *yrrN* as *trhP1* and *trhP2*, respectively. To determine whether these two paralogs act redundantly to form ho^5^U34, we constructed the double-deletion strain ∆*trhP1*/∆*trhP2* and analyzed the modification status of tRNA. The same level of mo^5^U34 was detected in this strain as in the single-knockout strains (Fig. 2e), suggesting that both paralogs are involved in synthesizing about 50% of mo^5^U34 in the cell, and another pathway is required for the remainder, as also observed for formation of cmo^5^U34 in *E. coli*.

We then focused on *yrrM*, which is encoded within the same operon as *trhP1* and *trhP2* (Fig. 2d). *B. subtilis* YrrM has high sequence similarity to the AdoMet-dependent catechol *O*-methyltransferase that catalyzes methylation of the hydroxyl group on the aromatic ring, suggesting that this protein is a methyltransferase responsible for mo^5^U34 formation. As expected, mo^5^U34 in tRNA^Val1^ disappeared and was converted to ho^5^U34 in a ∆*yrrM* strain (Fig. 2e). We then generated recombinant YrrM protein and performed in vitro reconstitution of mo^5^U34 in *E. coli* tRNA^Thr4^ containing ho^5^U34, which had been isolated from a ∆*cmoB* strain. mo^5^U34 was successfully synthesized in the presence of both YrrM and AdoMet (Supplementary Fig. 4). The product was confirmed by CID analysis (Supplementary Fig. 4). Taken together, these findings indicate that YrrM is an AdoMet-dependent methyltransferase that converts ho^5^U34 to mo^5^U34 in *B. subtilis*. During the preparation of this manuscript, *B. subtilis yrrM* was demonstrated to be a ho^5^U34-methyltransferase and renamed *trmR*^44^.

### Alternative pathway for tRNA hydroxylation

**Fig. 3:**
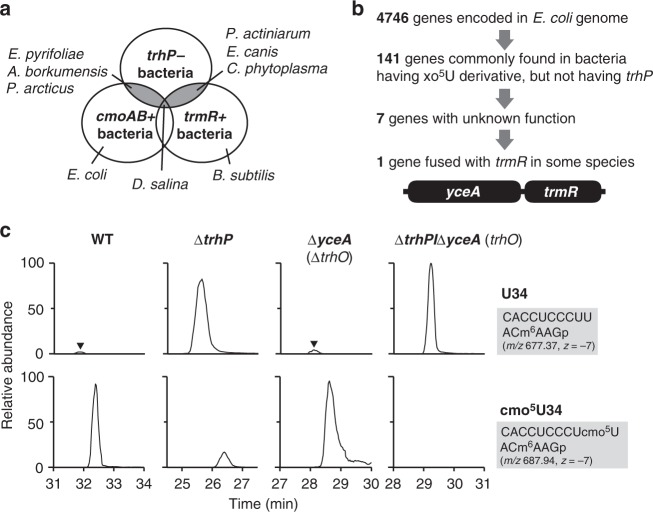
Identification of *trhO* responsible for tRNA hydroxylation. **a** Venn diagram depicts a group of organisms that have *cmoAB* or *trmR* homologs but no *trhP* homologs. *Erwinia pyrifoliae*, *Alcanivorax borkumensis*, and *Psychrobacter arcticus* harbor *cmoAB* but not *trhP*; *Pontibacter actiniarum*, *Ehrlichia canis*, and *Candidatus phytoplasma* harbor *trmR* but not *trhP*; and *Dactylococcopsis salina* has both *cmoAB* and *trmR* but not *trhP*. **b** Comparative genomics approach used to narrow down the candidate gene that bypasses ho^5^U34 biogenesis. In *Phytophthora sojae*, *Phytophthora ramorum*, and *Phaeodatylum tricornutum*, *yceA* and *trmR* are fused as a single gene. **c** Mass-spectrometric analysis of the wobble modification in *E. coli* tRNA^Val1^ isolated from WT (left panels), ∆*trhP* (middle left panels), ∆*yceA* (middle right panels), and ∆*trhP*/∆*yceA* (right panels) strains. XICs show anticodon-containing fragments of tRNA^Val1^ with U34 (upper panels) and cmo^5^U34 (lower panels). Black arrowheads indicate peaks corresponding to U34-containing fragments detected in the WT and ∆*yceA*. Sequence, *m/z* value, and charge state of each fragment are shown on the right

*E. coli trhP* and *B. subtilis trhP1*/*trhP2* are partially involved in the initial step of cmo^5^U34 and mo^5^U34 formation, respectively, indicating the existence of another redundant pathway for ho^5^U34 formation in both species. To search for the gene(s) responsible for this pathway, we used a comparative genomic approach. In bacteria, we found several organisms with *cmoA* and *cmoB* homologs, but no *trhP* homolog (Fig. 3a). Similarly, some bacteria had a *trmR* homolog, but no *trhP* homologs (Fig. 3a). Given that ho^5^U34 is a common precursor for cmo^5^U34 synthesis mediated by *cmoA* and *cmoB*, and mo^5^U34 synthesis mediated by *trmR*, the bacterial species lacking *trhP* homologs should have another gene responsible for ho^5^U34 formation independent of the *trhP* pathway. We identified seven bacterial species with *cmoAB* or *trmR* homologs but no *trhP* or *trhP1*/*trhP2* homologs (Fig. 3a). Among 4746 *E. coli* ORFs, we selected 141 genes (Fig. 3b) commonly present in all seven species, as well as in *B. subtilis*. We then narrowed down the list of candidates to seven genes with unknown functions (Fig. 3b). Among them, a *yceA* homolog was identified as a strong candidate because its genomic locus is close to that of *cmoA* in cyanobacteria, and *yceA* is encoded as a fusion protein with *trmR* in three bacterial species, *Phytophthora sojae*, *Phytophthora ramorum*, and *Phaeodatylum tricornutum* (Fig. 3b). To determine whether *yceA* is responsible for the second pathway of ho^5^U34 formation, we isolated tRNA^Val1^ from *E. coli* knockout strains of *trhP* and *yceA*, and analyzed the status of wobble modifications. The residual cmo^5^U34 observed in the Δ*trhP* strain completely disappeared in the double-deletion strain Δ*trhP*/Δ*yceA* (Fig. 3c), indicating that *yceA* is responsible for the second pathway of ho^5^U34. Hereafter, we refer to *yceA* as *trhO* (tRNA hydroxylation O). However, the amount of unmodified U34-containing fragment increased slightly in the single-deletion strain Δ*trhO* (Fig. 3c), indicating that the *trhP*-mediated pathway plays the predominant role in ho^5^U34 formation especially in the absence of *trhO*.

*B. subtilis ybfQ* is an ortholog of *E. coli trhO*. To determine whether *ybfQ* is involved in the second pathway for ho^5^U34 formation in *B. subtilis*, we constructed a triple-knockout strain, ∆*trhP1*/∆*trhP2*/∆*ybfQ*, and analyzed the status of tRNA wobble modifications. The modified mo^5^U34 in the ∆*trhP1*/∆*trhP2* strain, which was present at about 50% of the WT level, completely disappeared in the triple-deletion strain (Supplementary Fig. 5). However, as observed in the *E. coli* Δ*trhO* strain, little reduction in the mo^5^U34 level was observed in the single-deletion strain ∆*ybfQ* (Supplementary Fig. 5).

### Phylogenetic distribution of *trhP* and *trhO*

To investigate the phylogenetic distribution of *trhP*, *trhP1*, *trhP2*, and *trhO*, we generated a phylogenetic tree of organisms possessing or lacking each homolog (Supplementary Fig. 6, Supplementary Data 2). *trhO* orthologs predominated in bacteria and eukaryotes (40%; 232 of 584), whereas *trhP* orthologs (*yegQ* type in Supplementary Fig. 1) and *trhP1*/*trhP2* orthologs (PepU32#5/PepU32#3 in Supplementary Fig. 1) were only present in bacteria, and were less widely distributed [14% (84 of 584) and 5% (32 of 584), respectively] (Supplementary Fig. 6, Supplementary Data 2). *trhP* orthologs were mainly detected in γ- and β-proteobacteria, in addition to some desulfobacteria in δ-proteobacteria. *trhP1*/*trhP2* orthologs always co-occurred, and were present in phylum Firmicutes and some members of Tenericutes and Cyanobacteria. Given that TrhP1 is a related family with TrhP (Supplementary Fig. 1), TrhP1 might have branched out from TrhP, and evolved to require a paralogous protein TrhP2 that might be generated by gene duplication. Supporting this speculation, *trhP1* and *trhP2* are tandemly encoded in the same operon in *B. subtilis* (Fig. 2e). Also, we can explain the reason why *trhP* orthologs and *trhP1*/*trhP2* orthologs show a mutually exclusive distribution in bacteria (Supplementary Fig. 6). Intriguingly, over half of organisms bearing *trhP* or *trhP1*/*trhP2* orthologs also harbor *trhO* orthologs [49 of 84 (58%) organisms bearing *trhP*, *P* = 0.0003 (Fisher’s exact test); 22 of 32 (69%) organisms bearing *trhP1*/*trhP2*, *P* = 0.0007 (Fisher’s exact test)] (Supplementary Fig. 7). This significant overlap suggests that harboring both pathways for tRNA hydroxylation might help organisms to adapt to two different environments, i.e., aerobic and anaerobic conditions.

### Phenotypes of *E. coli* strains lacking tRNA hydroxylation

**Fig. 4:**
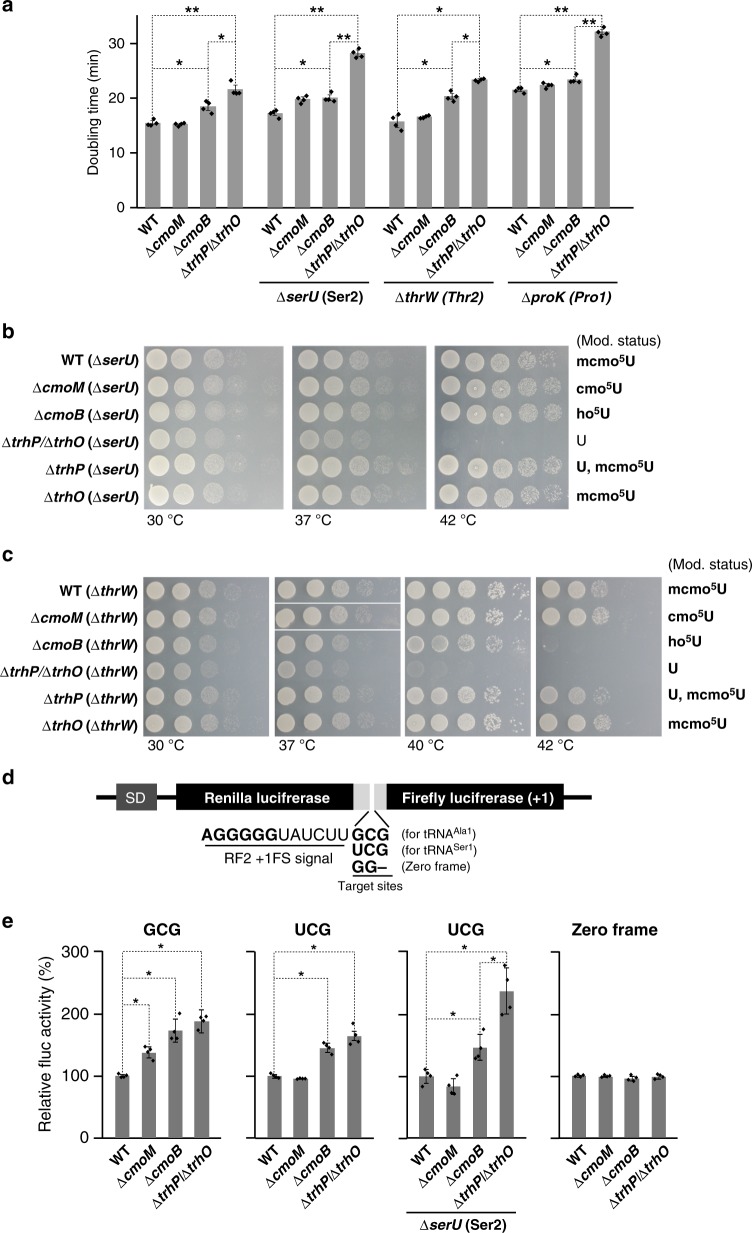
Phenotypes of *E. coli* strains lacking tRNA hydroxylation. **a** Growth rates of *E. coli* strains with different wobble modifications. Doubling time of WT (mcmo^5^U34 for tRNA^Ser1^, tRNA^Thr4^, and tRNA^Pro3^), ∆*cmoM* (cmo^5^U34), ∆*cmoB* (ho^5^U34), and ∆*trhP*/∆*trhO* (U34) in the presence (left) or the absence of tRNA isodecoders at 37 °C in liquid LB medium: tRNA^Ser2^ (middle left), tRNA^Thr2^ (middle right), or tRNA^Pro1^ (right). Individual data (dot plots) and their means ± SD (bar graph) are presented (*n* = 4). *P < 0.01; **P < 0.001 between two data series (Student’s *t*-test, one-sided). **b** Growth of WT, ∆*cmoM*, ∆*cmoB*, ∆*trhP*/∆*trhO*, ∆*trhP*, and ∆*trhO* strains in the absence of tRNA^Ser2^ (∆*serU*). The expected wobble modification status of each strain is shown on the right. Each strain was serially diluted (1:10), spotted onto LB agar plates, and cultivated for 11 h (30 °C), 8 h (37 °C) or 8 h (42 °C). **c** Growth of WT, ∆*cmoM*, ∆*cmoB*, ∆*trhP*/∆*trhO*, ∆*trhP*, and ∆*trhO* strains in the absence of tRNA^Thr2^ (∆*thrW*). The expected wobble modification of each strain is shown on the right. Each strain was serially diluted (1:10), spotted onto M9 minimum agar plates, and cultivated for 31 h (30 °C), 21 h (37 °C), 26 h (40 °C) or 31 h (42 °C). **d** Schematic of the reporter construct for the dual-luciferase assay, based on the RF2 recoding system. SD, Shine–Dalgarno sequence. *Renilla* and firefly luciferases were fused with a linker containing the +1 frameshift inductive signal of the RF2 recoding site. The frameshift target site was replaced with a GCG codon for tRNA^Ala1^, a UCG codon for tRNA^Ser1^, or GG (zero frame) for the control. **e** Relative pausing activities at the frameshift site with a GCG codon (left), a UCG codon in the presence (middle left) or absence (middle right) of isodecoder tRNA^Ser2^, or zero frame (right) were calculated by normalizing Fluc activity vs. Rluc activity and further normalizing against the WT activity in each graph. Individual data and their means ± SD (*n* = 4) are presented. *P < 0.01 between two data series (Student’s *t*-test, two-sided). All source data for Fig. 4 are provided as a Source Data file

We then measured the growth rate of a series of *E. coli* knockout strains involved in (m)cmo^5^U34 modifications. No growth reduction was observed in the ∆*cmoM* strain, as reported previously^13^ (Fig. 4a), indicating that the terminal methylation of mcmo^5^U34 has little impact on cell growth. A slight increase of doubling time was observed in the ∆*cmoB* strain, in which ho^5^U34 accumulated, indicating that the carboxymethyl group of (m)cmo^5^U34 contributes to efficient growth of *E. coli* cells. Notably, the ∆*trhP*/∆*trhO* strain grew more slowly than the WT and ∆*cmoB* strains, providing a clear evidence for the functional importance of 5-hydroxyl group of (m)cmo^5^U34 in cells. To characterize phenotypic features of the ∆*trhP*/∆*trhO* strain, we further knocked out tRNA genes responsible for G-ending codons [*serU* (tRNA^Ser2^) for the UCG codon, *thrW* (tRNA^Thr2^) for the ACG codon, and *proK* (tRNA^Pro1^) for the CCG codon], because these codons are redundantly deciphered by the respective tRNA and the isodecoder with the (m)cmo^5^U34 modification in each codon box. Growth reduction of the ∆*trhP*/∆*trhO* strain relative to the ∆*cmoB* strain was observed upon knockout of *serU*, *thrW*, and *proK* (Fig. 4a), indicating that the 5-hydroxyl group of (m)cmo^5^U34 plays a critical role in deciphering G-ending codons, especially in the absence of the respective isodecoder.

Next, we examined the temperature sensitivity of a series of knockout strains involved in (m)cmo^5^U34 modifications. The ∆*trhP*/∆*trhO/*∆*serU* strain grew as efficiently as the other strains on LB plates at 30 °C, but slowly at 37˚C and not at all at 42 °C (Fig. 4b). The growth defect of this strain at 42˚C was restored by introduction of plasmid-encoded *trhP* (Supplementary Fig. 8), indicating that the temperature-sensitive phenotype of this strain can be attributed to hypomodification of tRNA^Ser1^. Curiously, the ∆*trhP*/∆*serU* strain did not exhibit temperature sensitivity, although the frequency of mcmo^5^U34 actually decreased in tRNA^Ser1^ (Fig. 2c), indicating that *trhO*-mediated hydroxylation compensates for the growth defect when the *trhP*-mediated hydroxylation pathway is impaired. This result highlights the importance of redundant hydroxylation pathways for formation of xo^5^U. Similarly, we observed a severe growth reduction in the ∆*trhP*/∆*trhO/*∆*thrW* strain cultivated on M9 minimum plates, even at 40 °C (Fig. 4c). At 42 °C, this strain was unable to grow, and ∆*cmoB* strain exhibited a growth defect. These results clearly demonstrated that the absence of the 5-hydroxyl group of (m)cmo^5^U34 causes a temperature-sensitive phenotype, especially in the absence of the isodecoder responsible for G-ending codons.

### Decoding properties without tRNA hydroxylation

To investigate the functional role of (m)cmo^5^U34 modification in terms of decoding efficiency, we conducted dual-luciferase reporter assays based on the RF2 recoding system^18,45^. The reporter constructs consisted of *Renilla* luciferase (Rluc) fused with firefly luciferase (Fluc) in a +1 frame via a slippery linker derived from the +1 frameshift signal of the RF2 recoding site, so that Fluc expression requires a +1 frameshift at the linker sequence. The UGA codon at the recoding site was substituted with GCG and UCG as test codons to examine their ability to be decoded by tRNA^Ala1^ and tRNA^Ser1^, respectively (Fig. 4d). We also prepared a control reporter in which the recoding site was replaced with GG (zero frame). These reporters were introduced to a series of *E. coli* knockout strains lacking genes involved in (m)cmo^5^U34 modifications. The decoding ability of tRNAs with different wobble modifications at each test codon of the frameshift site was negatively correlated with the +1 frameshift activity, measured by Fluc activity, because in this system the +1 frameshift activity is promoted by the hungry A-site. The +1 frameshift activity was calculated by normalizing the Fluc signal against the Rluc signal (F/R value). No difference in F/R value in the control construct (zero frame) was observed in any strains (Fig. 4e).

Using this system, we first measured decoding ability of tRNA^Ala1^ with different wobble modifications using the GCG-reporter, because in *E. coli* the GCG codon is solely deciphered by this tRNA (Fig. 1b). Compared with the WT strain (mcmo^5^U34), we observed clear stimulation of +1 frameshift activity in ∆*cmoM* (cmo^5^U34) and ∆*cmoB* (ho^5^U34) strains (Fig. 4e), as reported previously^13^. We also observed a significant increase in +1 frameshift activity in the ∆*trhP*/∆*trhO* (U34) strain relative to the WT strain (mcmo^5^U34) (Fig. 4e). These results indicate that tRNA^Ala1^ gradually loses its decoding ability in response to side-chain shortening of mcmo^5^U34. Next, we measured the decoding ability of tRNA^Ser1^ with different wobble modifications using the UCG-reporter. In *E. coli*, UCG codons are redundantly decoded by tRNA^Ser1^ with the mcmo^5^UGA anticodon and tRNA^Ser2^ with the CGA anticodon (Fig. 1b). Relative to the WT strain (mcmo^5^U34), we observed a marked increase in +1 frameshift activity in the ∆*cmoB* (ho^5^U34) and ∆*trhP*/∆*trhO* (U34) strains (Fig. 4e). Then, we measured the activities of the UCG-reporter in a series of knockout strains in the absence of tRNA^Ser2^ (∆*serU* background). Relative to the ∆*serU* strain (mcmo^5^U34), +1 frameshift activity was elevated in both ∆*cmoB*/∆*serU* (ho^5^U34) and ∆*trhP*/∆*trhO*/∆*serU* (U34) strains (Fig. 4e). Notably, ∆*trhP*/∆*trhO*/∆*serU* (U34) had significantly higher +1 frameshift activity than ∆*cmoB*/∆*serU* (ho^5^U34) (Fig. 4e), indicating that the 5-hydroxyl group of ho^5^U34 on tRNA^Ser1^ can decode the UCG codon, especially in the absence of isodecoder tRNA^Ser2^.

### Characterization of *trhP*-mediated tRNA hydroxylation

**Fig. 5:**
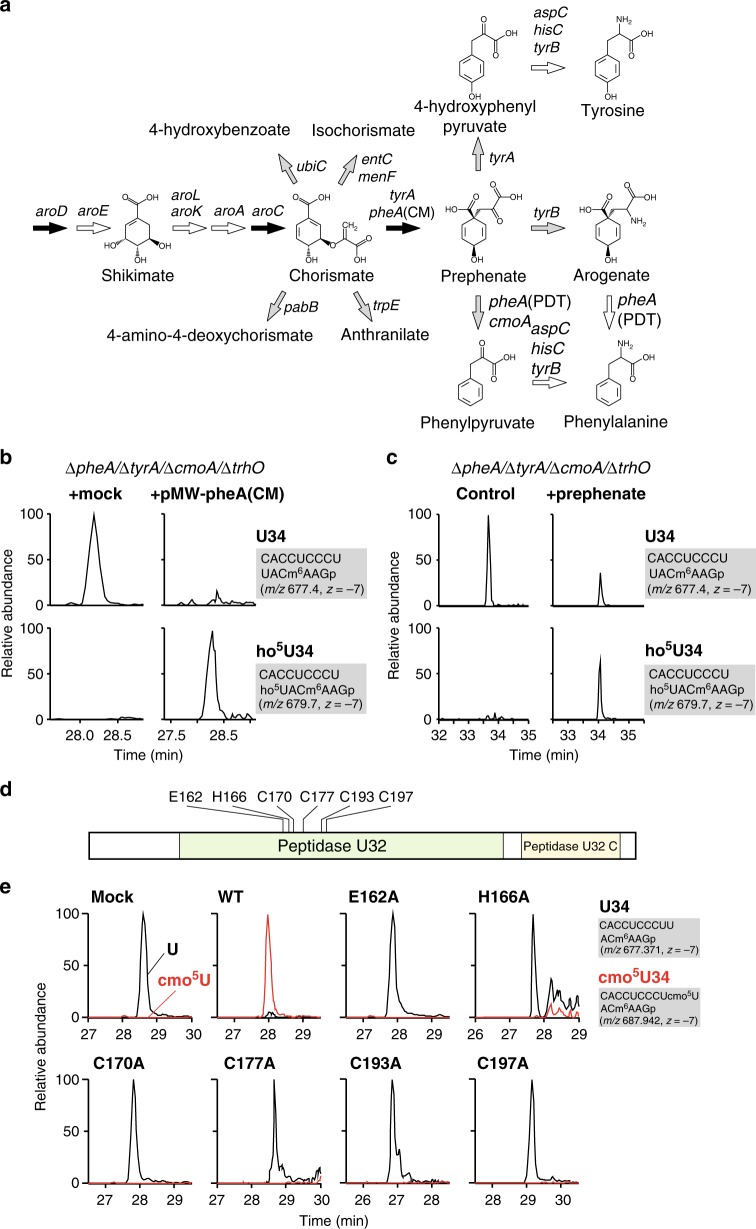
Characterization of *trhP*-mediated tRNA hydroxylation. **a** A shikimate pathway and related metabolism. Chemical structures of metabolites and the responsible genes (italicized) at each step are shown. Two or three genes at each step indicate a redundant pathway [e.g., prephenate is redundantly synthesized from chorismate mediated by *pheA*(CM) and *tyrA*]. Black or gray arrows represent pathways indispensable or dispensable for *trhP*-mediated ho^5^U34 formation, respectively (Supplementary Fig. 9). White arrows represent pathways not examined in this study. **b** Genetic complementation of ho^5^U34 formation. Mass-spectrometric shotgun analysis of total tRNAs obtained from the *E. coli* Δ*pheA*/Δ*tyrA*/Δ*cmoA*/Δ*trhO* strain transformed with a control plasmid (left panels) or pMW-*pheA*(CM) (right panels). XICs show multiply charged negative ions of anticodon-containing fragments of tRNA^Val1^ with U34 (upper panels) and ho^5^U34 (lower panels). Sequence, *m/z* value, and charge state of each fragment are shown on the right. **c** Metabolic complementation of ho^5^U34 formation. Mass-spectrometric shotgun analysis of total tRNAs obtained from the *E. coli* Δ*pheA*/Δ*tyrA*/Δ*cmoA*/Δ*trhO* strain cultured in the absence (left panels) or presence (right panels) of 1 mM prephenate. XICs show multiply charged negative ions of anticodon-containing fragments of tRNA^Val1^ with U34 (upper panels) and ho^5^U34 (lower panels). Sequence, *m/z* value, and charge state of each fragment are shown on the right. **d** Domain organization of *E. coli* TrhP, which contains Peptidase_U32 (PF01136) and Peptidase_U32_C (PF16325) domains. Six residues in the Peptidase_U32 domain that are essential for TrhP-mediated hydroxylation are indicated. **e** Mutation study of *trhP*. Mass-spectrometric shotgun analysis of total tRNA in the *E. coli* ∆*trhP* strain transformed with plasmid-encoded *trhP* WT or mutants, as indicated. XICs show multiply charged negative ions of the anticodon-containing fragments of tRNA^Val1^ with U34 (black lines) and cmo^5^U34 (red lines) in total tRNAs. Sequence, *m/z* value, and charge state of each fragment are shown on the right

According to our recent study^30^, the shikimate pathway is associated with *rlhA*-mediated ho^5^C2501 formation in 23S rRNA. A series of genetic studies revealed that prephenate is an essential metabolite for the first step of this modification. Given that TrhP belongs to a family of peptidase U32-containing proteins, we asked whether prephenate is also required for *trhP*-mediated ho^5^U34 formation. Consistent with this possibility, previous studies reported that the initial step of cmo^5^U34 formation is associated with chorismate biogenesis in *E. coli*, *B. subtilis*, and *Salmonella typhimurium*^20,46^. Chorismate is an end product of the shikimate pathway and a common precursor for aromatic amino acids and vitamins in bacteria and plants^47^. Shotgun analyses of *E. coli* total RNA revealed that cmo^5^U34 formation was significantly impaired in an ∆*aroC* strain, in which no chorismate was produced (Supplementary Fig. 9). However, ho^5^U34 was still present in this strain because ho^5^U34 was redundantly synthesized by the *trhO*-mediated pathway. By contrast, as expected, no ho^5^U34 was detected in an ∆*aroC*/∆*trhO* strain (Supplementary Fig. 10). In *E. coli*, chorismate is converted into five metabolites: isochorismate (catalyzed by the products of *entC* and *menF*), 4-hydroxybenzoate (*ubiC*), 4-amino-4-deoxychorismate (*pabB*), anthranilate (*trpE*), and prephenate (*pheA* and *tyrA*) (Fig. 5a). Among these pathways, the *ubiC*-mediated pathway was excluded because 4-hydroxybenzoate does not restore cmo^5^U34 formation in *Salmonella* ∆*aroD* strain^46^. As observed in the ∆*aroC* strain, cmo^5^U34 formation was only impaired in the ∆*pheA*/∆*tyrA* strain, but not in the ∆*entC*/∆*menF*, ∆*pabB*, and ∆*trpE* strains (Supplementary Fig. 9), indicating that prephenate or its downstream metabolites are required for ho^5^U34 formation.

Prephenate is converted to downstream metabolites via three pathways (Fig. 5a). cmo^5^U34 formation was unchanged in ∆*tyrA* and ∆*tyrB* strains (Supplementary Fig. 9). *pheA* encodes a fusion of chorismate mutase (CM) and prephenate dehydratase (PDT), which synthesize prephenate and phenylpyruvate, respectively (Fig. 5a). To dissect these two enzymatic activities, we constructed a *pheA* variant possessing only the CM activity [pheA(CM)] by introducing an active-site mutation in the PDT domain^48^. To determine whether prephenate is responsible for ho^5^U34 formation, we constructed the quadruple-knockout strain ∆*pheA*/∆*tyrA*/∆*cmoA*/∆*trhO*, and then introduced plasmid-encoded pheA(CM), resulting in accumulation of prephenate. ho^5^U34 levels were restored relative to those in a mock transformant (Fig. 5b). Furthermore, when prephenate was directly added to a culture medium of the quadruple-knockout strain, ho^5^U34 clearly appeared (Fig. 5c). These results demonstrated that prephenate is required for ho^5^U34 formation.

To characterize the peptidase U32 domain of *trhP*, we mutated each of six conserved residues in this domain (Fig. 5d, Supplementary Fig. 11) and examined their activities in vivo by complementation of the ∆*trhP*/∆*trhO* strain. Shotgun analyses revealed that cmo^5^U34 was fully restored by WT *trhP*, but not by any of the mutants examined in this study (Fig. 5e), indicating that the peptidase U32 domain plays a key role in *trhP*-mediated tRNA hydroxylation.

### Characterization of *trhO*-mediated tRNA hydroxylation

**Fig. 6:**
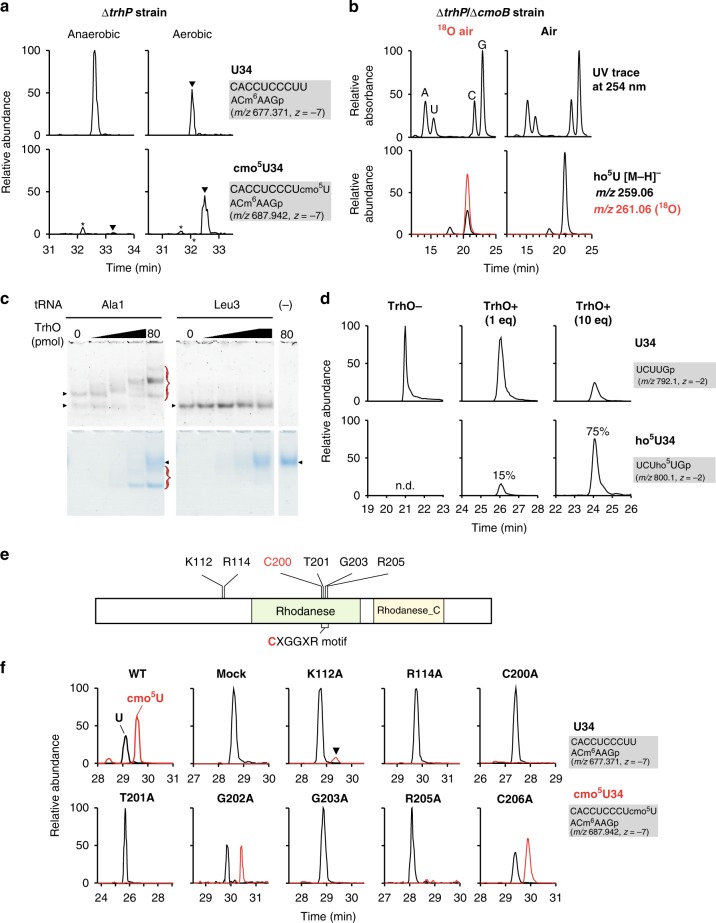
Characterization of *trhO*-mediated tRNA hydroxylation. **a** *trhO*-mediated cmo^5^U formation takes place under aerobic conditions. Mass-spectrometric shotgun analysis of total tRNAs obtained from *E. coli* Δ*trhP* strain cultured under anaerobic (left panels) or aerobic (right panels) conditions. XICs show negative ions of the anticodon-containing fragments of tRNA^Val1^ with U34 (upper panels) and cmo^5^U34 (lower panels). Arrowheads indicate target peaks, and asterisks indicate unspecific peaks. **b** Molecular O_2_ is the metabolic source of the hydroxyl group of ho^5^U34 generated by *trhO*. Mass-spectrometric nucleoside analyses of total RNAs obtained from the *E. coli* ∆*trhP*/∆*cmoB* strain cultured in mixed gas with 20% ^18^O_2_ (left panels) or in normal air (right panels). UV traces at 254 nm (upper panels) and XICs (lower panels) of deprotonated ho^5^U extracted from non-labeled (black line) or [^18^O]-labeled (red line) cells are shown. **c** TrhO interacts with tRNA^Ala1^ specifically. Electrophoretic mobility shift assay (EMSA) was performed to detect direct interaction between recombinant TrhO and tRNA^Ala1^ (left panels) or tRNA^Leu3^ (right panels) isolated from *E. coli*. tRNAs and TrhO were stained with SYBR Safe (upper panels) and Coomassie brilliant blue (lower panels), respectively. TrhO–tRNA complexes are indicated by red traces. Arrowheads indicate unbound intact tRNAs and TrhO. Source data are provided as a Source Data file. **d** In vitro reconstitution of ho^5^U34 with recombinant TrhO. RNase T_1_ digests of *E. coli* tRNA^Ser1^ transcripts (10 pmol) incubated with (10 pmol; 1 eq or 100 pmol; 10 eq) or without TrhO are subjected to RNA-MS. XICs show doubly charged negative ions of the anticodon-containing fragments of tRNA transcript with U34 (upper panels) and ho^5^U34 (lower panels). Frequencies of ho^5^U34 are indicated. **e** Domain organization of *E. coli* TrhO bearing Rhodanese (PF00581) and Rhodanese_C (PF12368) domains. The CXGGXR motif and six residues mutated in this study are indicated. **f** Mutation study of *trhO*. Mass-spectrometric shotgun analysis of total tRNAs in the *E. coli* Δ*trhP*/Δ*trhO* strain transformed with plasmid-encoded *trhO* WT or mutants, as indicated. XICs show multiply charged negative ions of the anticodon-containing fragments of tRNA^Val1^ with U34 (black lines) and cmo^5^U34 (red lines) from total tRNA

Given that Fe(II)- and 2-OG-dependent oxygenases use O_2_ as a substrate for hydroxylation, we next asked whether molecular oxygen is involved in ho^5^U34 formation. cmo^5^U34 formation is not affected in *E. coli* cultured under anaerobic conditions^20^, indicating that molecular oxygen is irrelevant to *trhP*-mediated ho^5^U34 formation. To determine whether O_2_ is involved in *trhO*-mediated ho^5^U34 formation, we cultured the *E. coli* ∆*trhP* strain under aerobic and anaerobic conditions, and then monitored cmo^5^U34 formation by shotgun analysis. Very little cmo^5^U34 was detected in ∆*trhP* cells cultured under anaerobic conditions (Fig. 6a), whereas no reduction in cmo^5^U34 was observed in WT cells (Supplementary Fig. 12), implying that O_2_ is necessary for *trhO*-mediated ho^5^U34 formation. We then metabolically labeled ho^5^U34 using ^18^O-labeled O_2_. To accumulate ho^5^U34, we cultured the *E. coli* ∆*trhP*/∆*cmoB* strain in mixed gas containing 20% ^18^O_2_. Total RNA extracted from this culture was digested into nucleosides and subjected to LC/MS. We clearly detected a deprotonated ho^5^U34 nucleoside with molecular mass (*m/z* 261) 2 Da greater than that of the naturally occurring nucleoside (Fig. 6b). CID analysis of the nucleoside revealed that the ^18^O atom was present in the base moiety of ho^5^U (Supplementary Fig. 13). No oxygens in the uracil base were labeled with ^18^O under the ^18^O-air condition (Supplementary Fig. 13), demonstrating that *trhO*-mediated tRNA hydroxylation utilizes O_2_ as an oxygen atom donor.

Next, we generated recombinant *E. coli* TrhO and subjected it to biochemical characterization. Electrophoretic mobility shift assay (EMSA) revealed that TrhO interacted with tRNA^Ala1^, but not with tRNA^Leu3^ (Fig. 6c). This result is consistent with our observation that cmo^5^U34 of tRNA^Leu3^ is mainly synthesized via the *trhP*-mediated pathway, whereas mcmo^5^U34 of tRNA^Ala1^ is redundantly synthesized by both the *trhP*- and *trhO*-mediated pathways. We then attempted to reconstitute ho^5^U in vitro, and successfully detected ho^5^U on tRNA^Ser1^ in the presence of recombinant TrhO. When TrhO was present in excess, 75% of the tRNA had ho^5^U (Fig. 6d). CID analysis of the modified fragment confirmed that ho^5^U was formed at position 34 of the tRNA (Supplementary Fig. 14). This result demonstrated that TrhO is an RNA hydroxylase that can form ho^5^U34 on tRNA by utilizing O_2_ as an oxygen donor.

TrhO is a rhodanese family protein characterized by the CXGGXR motif^49^, with an active-site cysteine in a glycine-rich loop (Fig. 6e, Supplementary Fig. 15). The crystal structure of *Legionella pneumophila* Lpg2838, a TrhO homolog, was solved by structural genomics (Supplementary Fig. 16)^50^. In the rhodanese domain, the active-site loop structure with the CTGGIR sequence (positions 200–205 in *E. coli* numbering) is formed by hydrogen bonds between T201 and R205 (corresponding to T178 and R182 in Supplementary Fig. 16). To characterize this motif, we constructed a series of *E. coli trhO* mutants in which C200, T201, G202, G203, R205, or C206 was mutated to alanine, and then examined their activities in vivo by complementation of the ∆*trhP*/∆*trhO* strain. The level of cmo^5^U34 in tRNA^Val1^ was partially restored by WT *trhO* (Fig. 6f). For the *trhO* mutants, C200, T201, G203, and R205 were essential for *trhO*-mediated tRNA hydroxylation, whereas G202 and C206 were not (fig. 6f). In the TrhO structure, a positively charged β-sheet is present near the active-site loop (Supplementary Fig. 16), providing a surface capable of recognizing tRNAs. The *trhO* mutants K112A and R114A, which lack positively charged residues on the β-sheet surface, hardly rescued or did not rescue cmo^5^U34 formation, respectively (Fig. 6f), suggesting that this surface is required for *trhO*-mediated tRNA hydroxylation.

## Discussion

In this study, we identified two independent pathways, mediated by *trhP* and *trhO*, involved in tRNA hydroxylation in the early steps of (m)cmo^5^U34 formation. We confirmed that (m)cmo^5^U34 was completely converted to unmodified U34 in a ∆*trhP*/∆*trhO* strain. This finding enabled us to analyze the physiological roles of (m)cmo^5^U34. This strain grew more slowly than a ∆*cmoB* strain, which has ho^5^U34, confirming the physiological importance of the O5 oxygen atom of (m)cmo^5^U34. In addition, the ∆*trhP*/∆*trhO* strain exhibited severe growth defects and temperature-sensitive phenotypes when each of the tRNA genes (*serU*, *thrW*, and *proK*) responsible for G-ending codons was simultaneously deleted. These genetic interactions strongly indicate that (m)cmo^5^U34 plays a functional role in efficiently deciphering G-ending codons. The luciferase reporter assay revealed that decoding of UCG was significantly impaired in the ∆*trhP*/∆*trhO* strain relative to the ∆*cmoB* strain in the absence of tRNA^Ser2^, suggesting that the ho^5^UGA anticodon decodes the UCG codon more efficiently than the UGA anticodon. These observations demonstrate the direct involvement of the O5 oxygen of the xo^5^U34 modification in codon recognition in vivo.

According to the crystal structure of the ribosome 30S subunit in complex with the anticodon-stem loop (ASL) of tRNA, cmo^5^U-G pairing forms Watson–Crick geometry (Supplementary Fig. 17), which is more stable than U-G wobble geometry due to the stacking interaction with the neighboring base pair (i.e., the second letters of codon and anticodon)^19^. The O5 oxygen of cmo^5^U34 makes a hydrogen bond with 2’ OH of U33 to pre-structure the ASL, presumably reducing entropic cost to base pair with any codons. In addition, the O5 oxygen may induce keto-to-enol tautomeric conversion of the uracil base to stabilize cmo^5^U-G pairing in Watson–Crick geometry. Moreover, because (m)cmo^5^U34 decodes G-ending codons more efficiently than ho^5^U34^13,18^, the carboxymethyl group and terminal methylation of (m)cmo^5^U34 contribute further to efficient codon recognition. Structural analysis has shown that the carboxymethyl group of cmo^5^U34 forms a hydrogen bond with the O4 carbonyl oxygen of U in the first letter of the codon, implying that the cmo^5^U side chain is directly involved in codon recognition^19^. Because ho^5^U34 has a phenolic hydrogen, and the pK_a_ value of O5 is 7.78^51^, ho^5^U34 is ionized to some extent under neutral pH conditions. Thus, the carboxymethylation of cmo^5^U34 and methylation of mo^5^U34 might confer efficient codon recognition by suppressing the ionization of ho^5^U34.

Our findings reveal that the TrhP-dependent pathway requires prephenate, whereas the TrhO-dependent pathway requires molecular oxygen. Thus, these two pathways are biochemically independent with respect to their requirement for metabolites. The existence of redundant and robust pathways for ho^5^U formation emphasizes that the xo^5^U34 modification is essential for bacteria to survive in a harsh environment. This is the unique instance of the RNA modification synthesized by two independent pathways in the same organism. According to phylogenetic distribution analysis in all domains of life (Supplementary Fig. 6), some organisms possess both *trhP* and *trhO* genes, whereas other organisms possess just one of them. Considering that anaerobes preceded aerobes in the early evolution on Earth, the *trhP* pathway might have been established in anaerobic bacteria before the *trhO* pathway arose, assuming that xo^5^U34 was present in such ancestral organisms. Presumably, the *trhO* pathway was acquired by aerobic bacteria after the O_2_ concentration increased on Earth. The *trhP* pathway is required for anaerobic bacteria, whereas the *trhP* and *trhO* dual pathways are useful for organisms that live in both anaerobic and aerobic environments.

TrhP is a peptidase U32-containing protein. Phylogenetic analysis has shown that peptidase U32-containing proteins can be classified into 12 subfamilies (Supplementary Fig. 1)^30^. We showed previously that three of these families include the RlhA proteins (RlhA1, RlhA2a, and RlhA2b) responsible for ho^5^C formation in 23S rRNA^30^. It is plausible that other subfamilies are also involved in hydroxylation of RNA or other biomolecules. *Clostridia* species harbor a member of the PepU32#1 family and *trmR*, but no homologs of *trhO* or *trhP*, implying that mo^5^U34 is present and that PepU32#1 family proteins are functional homologs of *trhP* in these species. *Helicobacter pylori*, a representative of the ε-proteobacteria, possesses a PepU32#2 family protein (HP0169), *cmoA*, and *cmoB*, but no homologs of *trhO* or *trhP*, indicating that cmo^5^U34 is present and suggesting that HP0169 is responsible for prephenate-dependent ho^5^U34 formation in this species. Intriguingly, HP0169 is required for gastric colonization by *H. pylori*^52^. Similarly, in *Salmonella enterica*, a *trhP* ortholog is associated with chicken macrophage infection^53^. Together, these findings suggest that xo^5^U34 contributes to bacterial infection and pathogenesis.

We found that prephenate is required for *trhP*-dependent ho^5^U34 formation. Given that prephenate is also a substrate for cmo^5^U34 formation mediated by CmoA and CmoB^21^, it is a critical metabolite involved in the entire pathway of cmo^5^U34 biogenesis. Prephenate is generated from chorismate, which in turn is a common precursor of multiple metabolites, including aromatic amino acids, quinones, folate, and siderophores^47^. Thus, cmo^5^U34 modification might be tightly associated with the shikimate pathway and biogenesis of aromatic amino acids. The frequency of cmo^5^U34 might be regulated by the cellular concentration of prephenate under some environmental stress conditions.

TrhP is a paralog of RlhA in the same family of peptidase U32-containing proteins. RlhA is responsible for prephenate-dependent ho^5^C2501 formation in *E. coli* 23S rRNA^30^, strongly implicating the involvement of the peptidase U32 motif in the C5-hydroxylation of pyrimidine base. Here, we showed that three conserved residues (E162, C170, and C177) in the motif of TrhP are essential for ho^5^U34 formation. Additionally, the corresponding residues (E161, C169, and C176) in RlhA were also required for ho^5^C2501 formation^30^, demonstrating that the peptidase U32 motif is directly involved in the hydroxylation of RNA. To date, we have no evidence that TrhP and RlhA directly catalyze the hydroxylation of RNA molecules. Given that RlhA is directly bound to the 50S subunit and its precursor in the cell, RlhA might be the hydroxylase responsible for ho^5^C2501 formation. By analogy, TrhP might be a hydroxylase for tRNA. Regarding the role of prephenate in ho^5^U34 formation, several possibilities should be considered. Prephenate might serve as an oxygen donor for ho^5^U34 formation, or alternatively as a coenzyme for the reaction. Moreover, we cannot exclude the possibility that unknown metabolites derived from prephenate are involved in ho^5^U34 formation. Further studies are necessary to elucidate the molecular mechanism underlying ho^5^U34 formation mediated by TrhP and prephenate.

*trhO* homologs are present in many aerobes and facultative anaerobes, but not in obligate anaerobes such as *Bacteroides*, *Clostridium*, and *Bifidobacterium*. *trhO* homologs are distributed in a wide range of bacteria, including α-, β-, and γ-proteobacteria, Bacilli, actinobacteria, the FCB group, cyanobacteria, and a subset of phylum Tenericutes (Supplementary Fig. 6). Intriguingly, *trhO* homologs are also widely distributed in vertebrates and other eukaryotes. This finding suggests the presence of an xo^5^U-type modification in eukaryotes.

We also showed that TrhO directly catalyzes oxygen-dependent ho^5^U34 formation. TrhO is related to rhodanese, which is involved in persulfide formation during detoxification of cyanide; however, the functions of most rhodanese family proteins remain unclear. In the context of RNA modifications, Tum1p is a rhodanese protein that mediates a persulfide sulfur for 2-thiouridine synthesis in eukaryotes^38^. Bacterial YbbB (also known as MnmH) is another rhodanese family protein responsible for biogenesis of 2-selenouridine (se^2^U)^54^ and geranyl-2-thioudirine (ges^2^U)^55^. Mutation study of TrhO revealed that the active-site loop of the rhodanese domain is responsible for ho^5^U34 formation, suggesting that the rhodanese domain plays a critical role in hydroxylation of uracil base. Future studies should seek to clarify the mechanism by which rhodanese catalyzes this reaction.

**Fig. 7:**
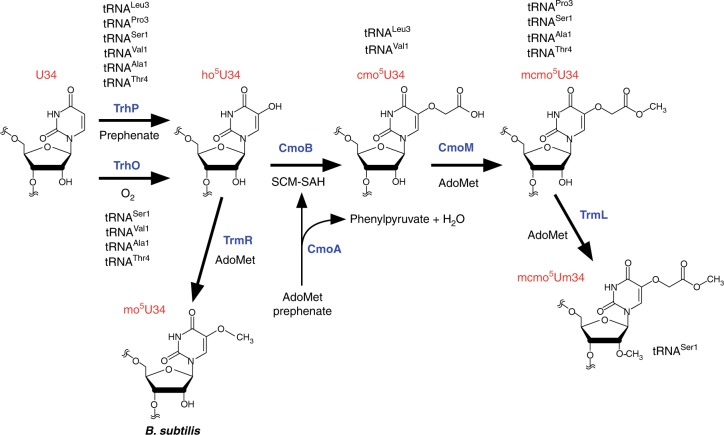
Biogenesis of mcmo^5^U34 and derivatives. At the first step, U34 is redundantly hydroxylated by independent pathways mediated by TrhP and TrhO to form ho^5^U34 in tRNAs responsible for decoding NYN codons. TrhP requires prephenate as a metabolite for ho^5^U34 formation, whereas TrhO uses O_2_ to hydroxylate U34 to form ho^5^U34 under aerobic conditions. CmoA synthesizes SCM-SAH from prephenate and AdoMet. CmoB uses SCM-SAH for carboxymethylation of ho^5^U34 to yield cmo^5^U34. Four tRNAs (for Ala1, Ser1, Pro3, and Thr4) are further methylated by CmoM to yield mcmo^5^U34. In Gram-positive bacteria, including *B. subtilis*, ho^5^U34 is methylated by TrmR to yield mo^5^U34

We now have a complete picture of xo^5^U34 formation in bacteria (Fig. 7). In the first step, U34 is redundantly hydroxylated by TrhP and TrhO to form ho^5^U34 in tRNAs responsible for decoding NYN codons. TrhP requires prephenate as a metabolite for ho^5^U34 formation, whereas TrhO uses a molecular oxygen for this purpose under aerobic conditions. In *E. coli*, TrhP is involved in ho^5^U34 formation of all six tRNA species, but has a preference for tRNA^Leu3^ and tRNA^Pro3^, whereas TrhO mainly hydroxylates the other four species. CmoA employs prephenate and AdoMet to generate SCM-SAH, a metabolite used for carboxymethylation of ho^5^U34 to yield cmo^5^U34 catalyzed by CmoB. Four tRNAs (for Ala1, Ser1, Pro3, and Thr4) are further methylated by CmoM to yield mcmo^5^U34. mcmo^5^U34 in tRNA^Ser1^ is partially methylated by TrmL to yield mcmo^5^Um34 as a minor modification. In Gram-positive bacteria, including *B. subtilis*, ho^5^U34 is methylated by TrmR to yield mo^5^U34 instead of (m)cmo^5^U34.

## Methods

### Strains and media

A series of single-knockout strains of *E. coli* and their parent strain were obtained from the National BioResource Project (NBRP), National Institute of Genetics (NIG), Japan (Keio collection)^56^. Other knockout strains were generated by homologous recombination using λ-derived Red recombinase^57^ with the chloramphenicol-resistance (Cm^R^) or the kanamycin-resistance marker (Kan^R^); all strains were selected with the appropriate antibiotics (20 µg per ml chloramphenicol or 50 µg per ml kanamycin). *E. coli* strains with multiple gene deletions were constructed by P1 transduction. The Kan^R^ marker was removed by pCP20 transformation^56^. Genotyping of each construct was performed by colony PCR. A series of knockout strains [Δ*yrrO* (Δ*trhP1*)::Em^R^, Δ*yrrN* (Δ*trhP2*)::Em^R^, and Δ*ybfQ* (Δ*trhO*)::Em^R^] and their parent strain [*B. subtilis* 168 MGNA-A001 (*trpC2*)] were obtained from the NBRP, NIG, Japan^58^. To construct Δ*yrrN*/Δ*yrrO*, Δ*yrrM*, and Δ*ybfQ*/Δ*yrrN*/Δ*yrrO* strains, the Cm^R^ cassette amplified from pDLK2 (courtesy of Akiko Soma, Chiba Univ.) was flanked by the 5’ and 3’ regions (~800 bps) of the target genes by ligation PCR, followed by transformation and selection with 5 µg per ml chloramphenicol. All constructs were confirmed by colony PCR. All primers and strains used in this study are listed in Supplementary Data 3 and Supplementary Table 2, respectively.

M9 minimal medium with 0.4% glucose or LB medium, as well as the corresponding solid media containing 1.5% agar, were used for growth analysis of *E. coli*. MOPS medium with 20 amino acids (10 mM Ser, 0.8 mM Ala/Gly/Leu, 0.6 mM Gln/Glu/Val, 0.4 mM Arg/Asn/Ile/Lys/Phe/Pro/Thr, 0.2 mM His/Met/Tyr, and 0.1 mM Cys/Trp), five vitamins (0.02 mM thiamine hydrochloride, 0.02 mM calcium pantothenate, 0.02 mM 4-aminobenzoic acid, 0.02 mM 4-hydroxybenzoic acid, 0.02 mM 2,3-dihydroxybenzoic acid), 0.4% glucose, and appropriate antibiotics, in the presence or the absence of 1 mM prephenate (Sigma-Aldrich, St. Louis, MO, USA), was used to determine the metabolite required for the *trhP* pathway.

### Plasmid construction

For the genetic rescue study, CDSs of *trhP*, *trhO*, and *pheA* with their 200 bp upstream sequences (including native promoter regions) were PCR-amplified from the *E. coli* BW25113 genome and cloned into pMW118 (Nippon Gene) to yield pMW-trhP, pMW-trhO, and pMW-pheA, respectively. pMW-pheA(CM), which lacks prephenate dehydratase activity due to the T278A mutation^48^, and a series of point mutants of pMW-trhP and pMW-trhO were constructed by QuikChange site-directed mutagenesis (Agilent Technology). For expression vectors of TrhO (YceA) and TrmR (YrrM), CDSs of *trhO* and *trmR* were PCR-amplified from genomic DNAs of *E. coli* BW25113 and *B. subtilis* 168, respectively, and cloned into pET21b (Novagen) to yield pET-trhO and pET-trmR. All constructs were confirmed by Sanger sequencing. All primers used in this study are listed in Supplementary Data 3.

### RNA extraction and tRNA isolation

Total RNA from each *E. coli* strain was extracted by phenol under acidic conditions^35^. The cells suspended in 1 × TE buffer [10 mM Tris (pH 8.0), 1 mM EDTA] was mixed with an equal volume of water-saturated phenol, followed by freeze and thaw twice and vigorous mixing for one hour at room temperature. The aqueous phase was separated by centrifugation, transferred to a new tube, washed with chloroform, and further purified using TRIzol reagent (Thermo Fisher Scientific, Waltham, MA, USA) and chloroform. RNA was recovered by 2-propanol precipitation. Individual tRNAs were isolated by reciprocal circulating chromatography (RCC)^42^ using a series of DNA probes^13^ (Supplementary Data 3). For RNA-MS shotgun analyses^13^, *E. coli* strains were cultured overnight, harvested, and resuspended in TE buffer to extract total RNA by TRIzol. The small RNA fraction was obtained as follows^13^. 50–250 μg of total RNA in 800 μl of 3 M NH_4_OAc (pH 5.3) was mixed with 640 μl (0.8 volume) of isopropanol at room temperature and centrifuged at 20,400×*g* for 10 min to precipitate long RNAs including rRNAs. The smaller RNA fraction was precipitated with ethanol from the supernatant. For RNA-MS shotgun analyses of *B. subtilis* tRNA modifications, the harvested cells were treated with 1 mg per ml lysozyme for 10 min on ice, and then subjected to TRIzol treatment to extract total RNA.

### Mass spectrometry of tRNA modifications

Liquid chromatography–mass spectrometry (LC/MS) analyses of tRNA modification were conducted as follows^13,35^. For RNA fragment analyses of individual tRNAs and shotgun analyses, 1.25 pmol of tRNA or 50 ng of the small RNA fraction was digested with 50 units RNase T_1_ (Thermo Scientific) in 20 mM NH_4_OAc (pH 5.3), followed by addition of an equal volume of 0.1 M triethylamine acetate (TEAA; pH 7.0). The RNase T_1_ digests were subjected to the trap column for desalting and chromatographed by HiQ sil C18W-3 nanospray column (C18, 3μm, 120 Å pore size, ID 0.1 × 100 mm, KYA technologies) with solvent system consisted of 0.4 M 1,1,1,3,3,3-hexafluoro-2-propanol (HFIP) (pH 7.0) (solvent A) and 0.4 M HFIP (pH 7.0) in 50% methanol (solvent B) at a flow rate of 300 nl per min with a linear gradient of 5–100% B solvent over 35 min with a splitless nano HPLC system (DiNa, KYA Technologies). The eluent was ionized by ESI source and introduced into an ion trap-orbitrap hybrid mass spectrometer (LTQ Orbitrap XL, Thermo Fisher Scientific). Ions were scanned with a negative polarity mode over an *m/z* range of 600–2000 throughout the separation. XICs were plotted according to the theoretical *m*/*z* of each fragment (Supplementary Table 1). LC/MS nucleoside analysis was performed using Q Exactive Hybrid Quadrupole-Orbitrap Mass Spectrometer (Thermo Fisher Scientific) equipped with a Dionex UltiMate 3000 LC System (Thermo Fisher Scientific) essentially as described^13,59^. Twenty micrograms of the small RNA fraction were digested at 37 °C for 3 h in 60 µl of solution containing 20 mM trimethylamine-HCl (TMA-HCl) (pH 7.0), 0.05 U of nuclease P1, and 0.1 U of BAP. The digests mixed with acetonitrile (f.c. 90%) were chromatographed by ZIC-cHILIC column (3 μm particle size, 2.1 × 150 mm, Merck Millipore) with a guard column with the same resin (2.1 × 20 mm, Merck Millipore) using a solvent system consisted of 5 mM NH_4_OAc (pH 5.3) (solvent A) and acetonitrile (solvent B) at a flow rate of 100 μl min^−1^ in a multistep linear gradient; 90–40% B from 0 to 30 min, 40% B for 10 min, and then initialized to 90% B. The eluent was directly introduced into the ESI source and analyzed by mass spectrometer. Deprotonated nucleosides were scanned in a negative polarity mode over a *m/z* range of 110–700 throughout separation. Data were analyzed using Xcalibur software (Thermo Fisher Scientific).

Basically, in negative mode of ESI, ionization efficiencies of the RNA fragments bearing the same sequence but different modification do not differ largely, because ESI ionization relies mainly on numbers of phosphate groups, not on type of base modifications^60^. Thus, we relatively quantified modification frequencies of RNA fragments with different chemical structures from their intensities of XICs.

### Luciferase reporter assay

The dual-luciferase reporter assay^13^ was performed as follows: knockout strains were transformed with pBAD-RFlucGCG, pBAD-RFlucUCG, or pBAD-RFlucGG. Each transformant was pre-cultivated at 37 °C in 2 ml LB medium containing 100 µg per ml ampicillin overnight. The preculture (1 ml) was inoculated to 2 ml of LB medium containing 100 µg per ml ampicillin and 100 µM arabinose to induce expression of the reporter. When the OD_600_ reached to 0.3–0.7, 1 ml aliquot was centrifuged, and the pelleted cells were resuspended in 200 µl of lysis buffer [10 mM HEPES-KOH (pH 7.5), 100 mM NaCl, 10 mM MgCl_2_, 7 mM 2-mercaptoethanol, 400 µg per ml lysozyme]. Cell lysates were prepared by the freeze–thaw and cleared by centrifugation. The luciferase reporter assay was performed with 5 μl lysate using GloMax™ 96 Microplate Luminometer (Promega). The Fluc luminescence signal was normalized against the Rluc signal.

### Anaerobic cultivation

WT and Δ*trhP* strains were precultured in a BioShaker G·BR-200 (TAITEC) at 37 °C with rotation at 60 rpm for 24 h in 10 ml of degassed LB medium in a 10 cm dish doubly packed in Ziploc (Asahi-Kasei, Japan) with one bag of AnaeroPack-Anaero (Mitsubishi Gas Chemical, Japan) and an oxygen indicator (OXY-1, JIKCO). The preculture (100 µl) was inoculated into 10 ml of degassed LB medium, sealed with an AnaeroPack-Anaero, incubated at room temperature for 1 h to deoxidize completely, and then cultivated at 37 °C overnight at 60 rpm.

### Metabolic labeling analysis using ^18^O_2_

^18^O-containing mixed gas [20% ^18^O_2_ (97.5% ^18^O_2_, 1% ^17^O_2_, 1.5% ^16^O_2_), 80% N_2_] was obtained commercially (Tatsuoka, Japan). The Δ*cmoB*/Δ*trhP* stain was precultured at 37˚C overnight in LB medium containing 50 µg per ml kanamycin. The preculture (1 ml) was inoculated into 100 ml of degassed LB medium containing 1 mM uridine and 50 µg/ml kanamycin packed in a PVDF air-sampling bag (As One, Japan). Inside air was carefully removed, and then replaced once with N_2_ and twice with ^18^O_2_ mixed gas. The bag was capped and sealed with Parafilm (Bemis), and then cultured at 37 °C for 24 h at 100 rpm in a BioShaker G·BR-200 (TAITEC). Total RNA was extracted from the culture, digested into nucleosides, and analyzed by LC/MS as described above.

### Preparation of recombinant protein

*E. coli* BL21(DE3) transformed with pET-trhO was cultured at 37 °C until OD_600_ reached ~0.7, supplemented with 0.4% lactose, and further cultured at 16 °C for 32 h. The *E. coli* Rosetta(DE3) transformed with pET-trmR was cultured at 37 °C until OD_600_ reached ~0.7, supplemented with 100 µM IPTG, and further cultivated at 37 °C for 4 h to induce overexpression. Cells were sonicated in lysis buffer for TrhO [50 mM HEPES-KOH (pH 7.5), 300 mM KCl, 10 mM MgCl_2_, 1 mM dithiothreitol (DTT), and 0.2 mM PMSF] or the lysis buffer for TrmR [50 mM HEPES-KOH (pH 7.5), 100 mM KCl, 10 mM MgCl_2_, 1 mM dithiothreitol (DTT), and 0.2 mM PMSF]. The recombinant proteins TrhO and TrmR were purified using a HisTrap column (GE Healthcare) with a linear gradient of imidazole (0–500 mM for TrhO, 25–500 mM for TrmR). Purified protein was dialyzed in the individual lysis buffer, supplemented with glycerol to a final concentration of 30%, and stored at −20 °C.

### In vitro reconstitution of tRNA modification

For in vitro ho^5^U formation by TrhO, tRNA^Ser1^ transcript (10 pmol) and recombinant TrhO (10 or 100 pmol) were incubated at 37 °C for 1 h in a reaction mixture (10 µl) containing 25 mM Tris-HCl (pH 7.0), 300 mM NaCl, 1 mM MgCl_2_, and 10 mM 2-mercaptoethanol. The tRNA was extracted with acidic phenol/chloroform and precipitated with ethanol, followed by RNase T_1_ digestion and RNA-MS analysis as described above.

In vitro methylation of ho^5^U by TrmR was performed essentially as described^13^. *E. coli* tRNA^Thr4^ bearing ho^5^U34 was isolated from the Δ*cmoB* strain. The reaction mixture (10 µl) containing 10 pmol of tRNA^Thr4^, 20 pmol of TrmR, 50 mM HEPES-KOH (pH 7.5), 100 mM KCl, 10 mM MgCl_2_, and 7 mM 2-mercaptoethanol was incubated at 37 °C for 1 h in the presence or absence of 1 mM AdoMet.

### Electrophoresis mobility shift assay

EMSA was performed essentially as described^34^. Recombinant TrhO (0, 10, 20, 40, or 80 pmol) and in vitro transcribed tRNA^Ala1^ or tRNA^Leu3^ (20 pmol each) were incubated at 37 °C for 1 h in 10 µl of reaction mixture [50 mM HEPES-KOH (pH 7.5), 5 mM Mg(OAc)_2_, 100 mM KCl, 1 mM spermine, 1 mM DTT]. The mixtures were electrophoresed in 6% native polyacrylamide gel with running buffer [50 mM HEPES-KOH (pH 7.5), 5 mM Mg(OAc)_2_, and 1 mM DTT] in cold room. The gel was first stained with SYBR Safe (Thermo Fisher Scientific) to detect tRNA and then with Coomassie brilliant blue (Nacalai Tesque) to detect protein.

### Comparative genomics

The comparative genomics approach used to identify the *trhO* (*yceA*) gene was performed with the IMG database^61^. The gene occurrence profile was used to select seven organisms in which *cmoBA* or *yrrM* homologs were present and *trhP* (*yegQ*) or *trhP1/trhP2* homologs were absent: *Ehrlichia canis* str. Jake, *Erwinia pyrifoliae* DSM12163, *Alcanivorax borkumensis* SK2, *Candidatus Phytoplasma asteris* onion yellows OY-M, *Pontibacter actiniarum* sp. BAB1700, *Dactylococcopsis salina* PCC 8305, and *Psychrobacter arcticus* 273–4. Using the phylogenic profiler, 141 *E. coli* genes that are conserved in these seven organisms and *B. subtilis* were identified. According to the UniProt gene annotation, seven uncharacterized genes were picked as candidates.

### Phylogenetic analysis

The phylogenetic tree of peptidase U32 (Supplementary Fig. 1) was generated as described^30^. Species names matched to proteins were retrieved from UniProt. The occurrence profiles of *trhO*, *trhP*, *trhP1*, *trhP2*, *cmoA*, *cmoB*, *trmR*, and *cmoM* homologs (Supplementary Fig. 6) were retrieved from GTOP^62^ or the Interpro database^63^. To generate the phylogenetic tree, 584 organisms that are registered in the databases we used, i.e., species listed in GTOP, phyloT, and Pfam, were selected.

### Reporting summary

Further information on research design is available in the Nature Research Reporting Summary linked to this article.

## Supplementary information

Supplementary Information

Peer Review Files

Reporting Summary

Description of Additional Supplementary Files

Supplementary Data 1

Supplementary Data 2

Supplementary Data 3

Source Data

## Data Availability

A reporting summary for this Article is available as a Supplementary Information file. The source data for graphs and gels are provided as a Source Data file. All data is available from the corresponding author upon reasonable request.
