# Supplementary material for: Dual pathways of tRNA hydroxylation ensure efficient translation by expanding decoding capability
Source: Nat Commun. 2019 Jun 28;10:2858. doi: 10.1038/s41467-019-10750-8 (PMC6599085; doi:10.1038/s41467-019-10750-8)
Supplement: Supplementary file 1 — Supplementary Information [file 41467_2019_10750_MOESM1_ESM.pdf]

Supplementary information

Dual pathways of tRNA hydroxylation ensure efficient translation by expanding decoding capability

Yusuke Sakai[+], Satoshi Kimura[+], and Tsutomu Suzuki*

Department of Chemistry and Biotechnology, Graduate School of Engineering, University of Tokyo, 7-3-1 Hongo, Bunkyo-ku, Tokyo 113-8656, Japan

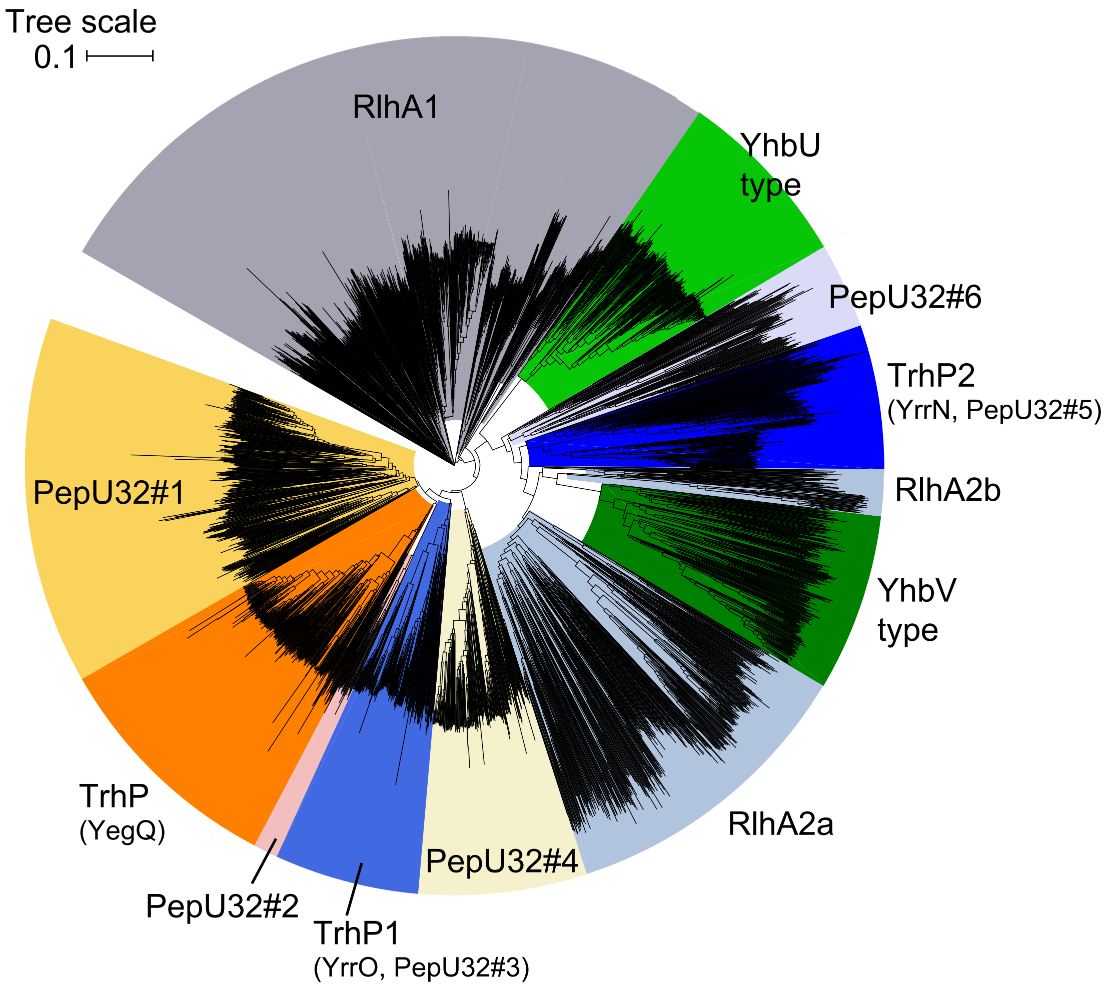

Supplementary Figure 1. Phylogenetic analysis of peptidase U32 families. Neighbor-joining tree of 3521 peptidase U32 motifs deposited in Pfam. Accession numbers and sources of all homologs are listed in Supplementary Data 1. The 12 subfamilies are shown in different colors.

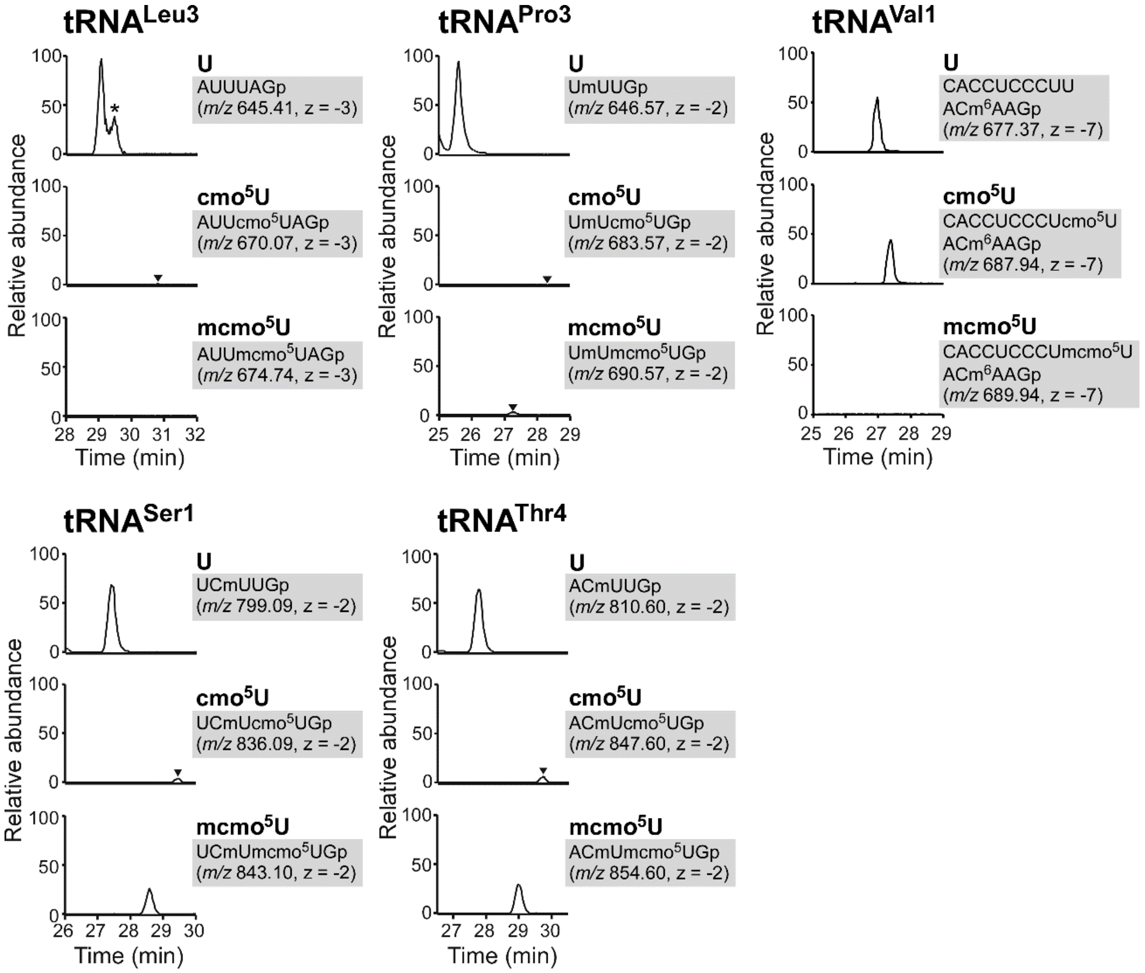

Supplementary Figure 2. Mass-spectrometric analyses of the wobble modifications of individual tRNAs isolated from the *yegQ* knockout strain.

XICs show the anticodon-containing fragments with U34 (top row of each panel), cmo^{5}U34 (middle row), and mcmo^{5}U34 (bottom row) from the indicated tRNAs isolated from the Δ*yegQ* strain. Arrowheads indicate target peaks. The asterisk indicates an unspecific peak. Sequence, *m/z* value, and charge state of each fragment are shown on the right.

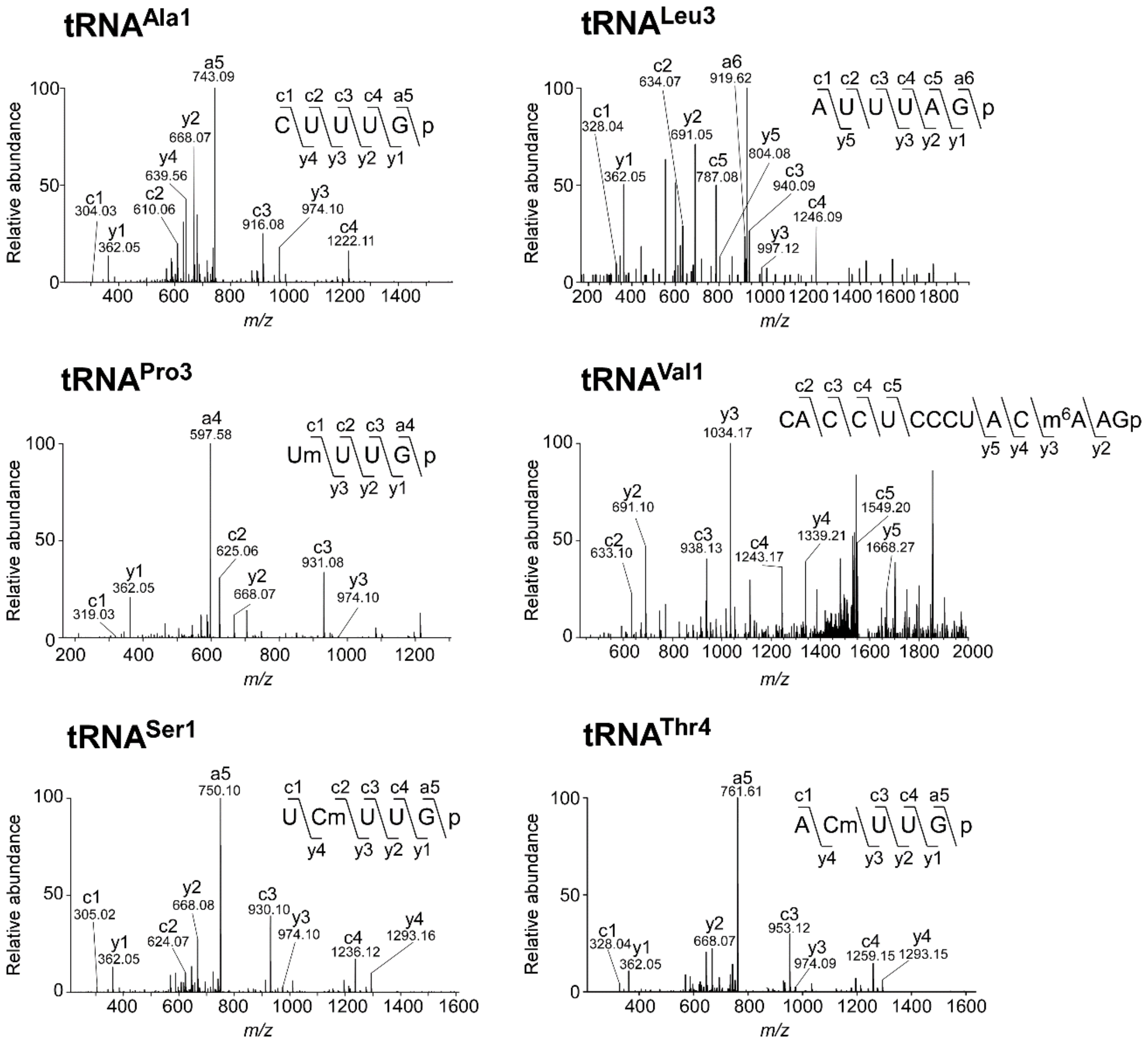

Supplementary Figure 3. Collision-induced dissociation (CID) spectra of the anticodon-containing fragments of tRNAs isolated from the Δ*yegQ* strain.

Multiply charged negative ions of the RNase T_1–digested fragments containing U34 from each tRNA were used as precursor ions for CID. The product ions were assigned as described previously [1]. The sequences of the parent ion and assigned product ions are described in each panel. These data confirm the presence of U34 in each tRNA.

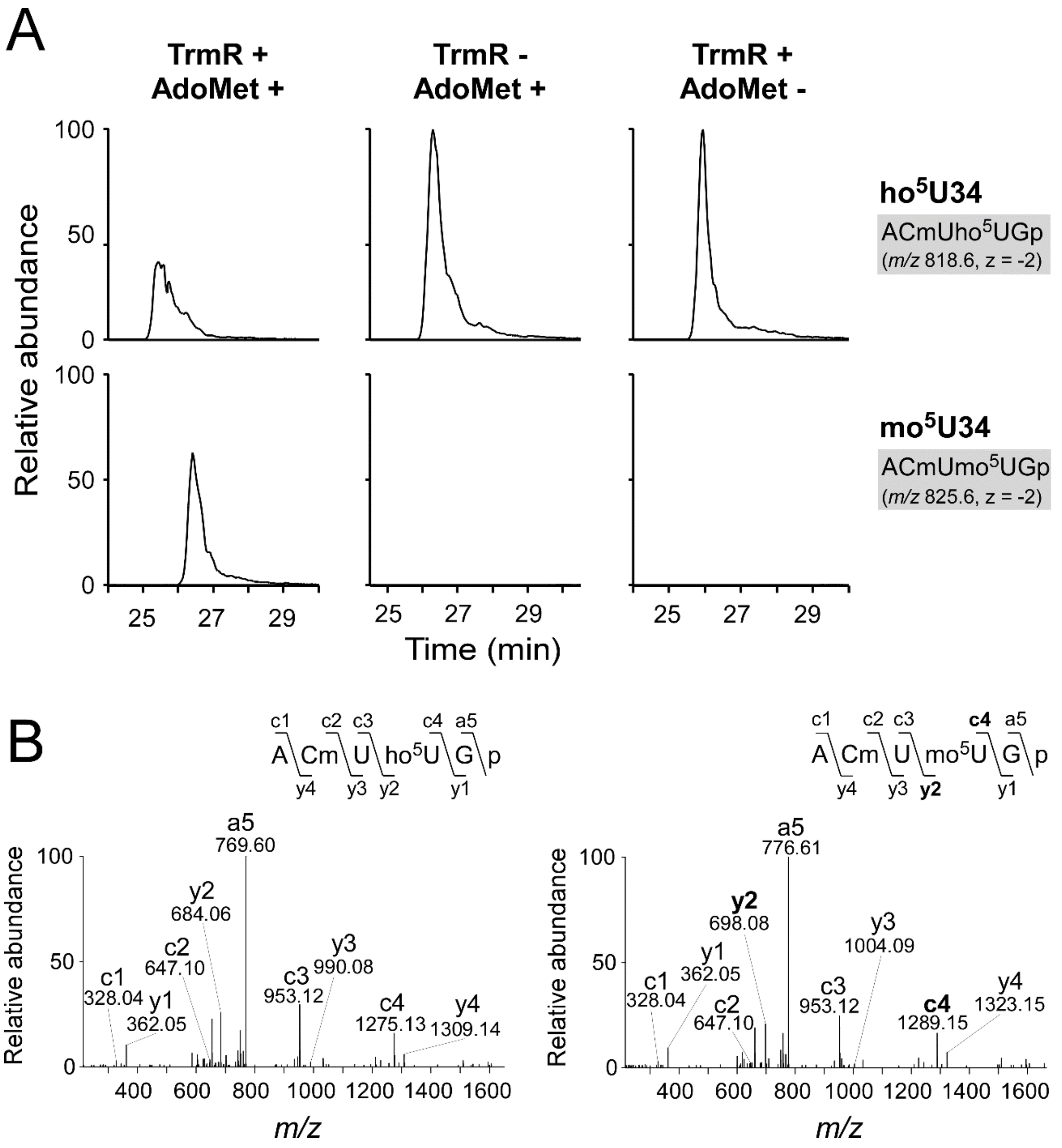

Supplementary Figure 4. ***In vitro*** **reconstitution of ho^{5}U methylation with recombinant TrmR.**

(A) *E. coli* tRNA^{Thr4} bearing ho^{5}U isolated from the Δ*cmoB* strain was incubated in the presence or absence of recombinant TrmR, with or without AdoMet. Top and bottom panels show XICs of the doubly charged negative ions of the RNase T$_1$–digested fragments containing ho^{5}U34 and mo^{5}U34, respectively.

(B) CID spectra of the RNase T$_1$–digested fragments containing ho^{5}U34 (left panel) and mo^{5}U34 (right panel). Product ions were assigned as described previously [1]. The sequences of the parent ion and assigned product ions are described in each panel.

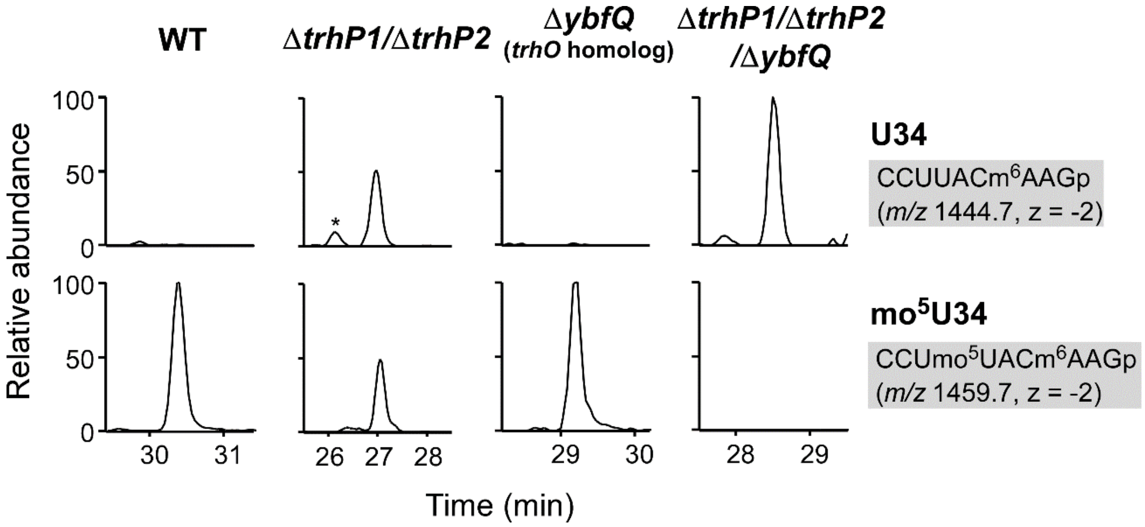

Supplementary Figure 5. ***B. subtilis ybfQ*** **is an ortholog of** ***E. coli trhO*****.**

RNA-MS shotgun analysis of mo^5U formation in *B. subtilis* strains. XICs show the doubly charged negative ions of the anticodon-containing fragments of $tRNA^{Val1a,b}$ with U34 (upper panels) and mo^5U34 (lower panels) from total tRNA isolated from the WT (left panels), Δ*trhP1*/Δ*trhP2* (middle left panels), Δy*bfQ* (middle right panels), and Δ*trhP1*/Δ*trhP2*/Δy*bfQ* (right panels). The sequence, *m/z* value, and charge state of each fragment are shown on the right. The asterisk indicates a nonspecific peak with the same *m/z* value.

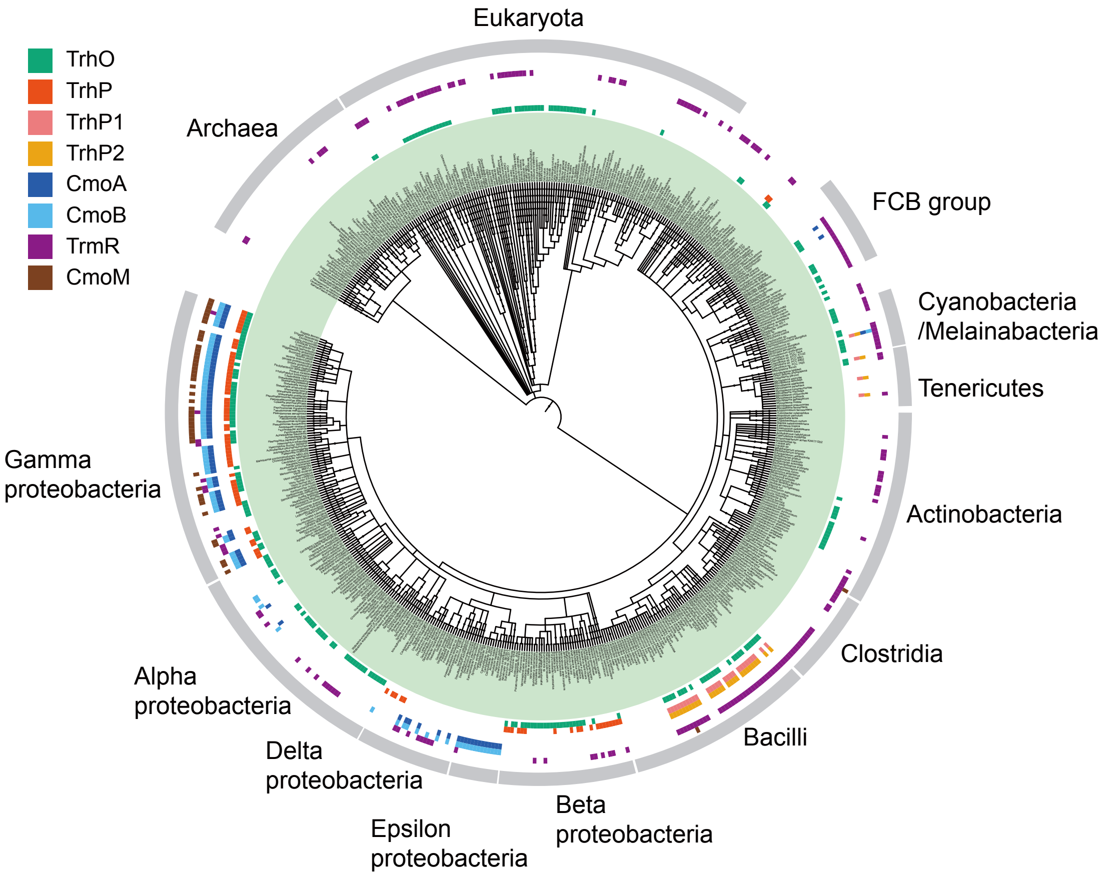

Supplementary Figure 6. Phylogenetic distribution of genes responsible for xo^{5}U synthesis.

A phylogenetic tree of 584 organisms from all three domains of life is displayed, with color strips indicating the distribution of TrhO (green), TrhP (red), TrhP1 (pink), TrhP2 (orange), CmoA (blue), CmoB (sky blue), TrmR (purple), and CmoM (brown). Phyla are also shown as gray strips. The list of organisms, along with the distributions of a series of genes, is also provided in Supplementary Data 2.

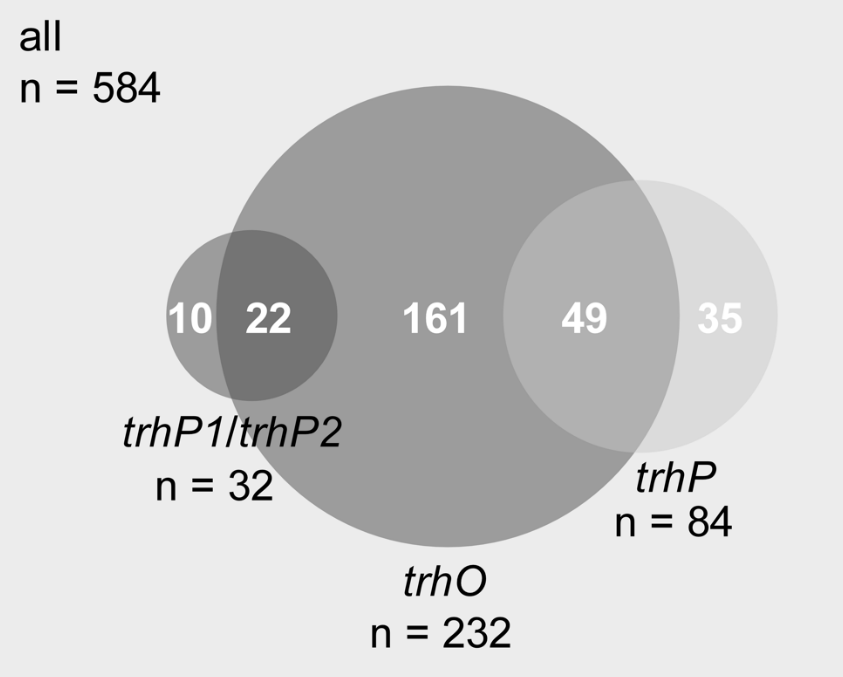

Supplementary Figure 7. Overlapping distributions of *trhO*, *trhP*, and *trhP1/trhP2*. The three circles indicate the numbers of species bearing *trhP1*/*trhP2* (n = 32), *trhO* (n = 232), and *trhP* (n = 84). Among them, 22 species harbor *trhO* and *trhP1*/*trhP2*, whereas 49 species harbor *trhO* and *trhP*.

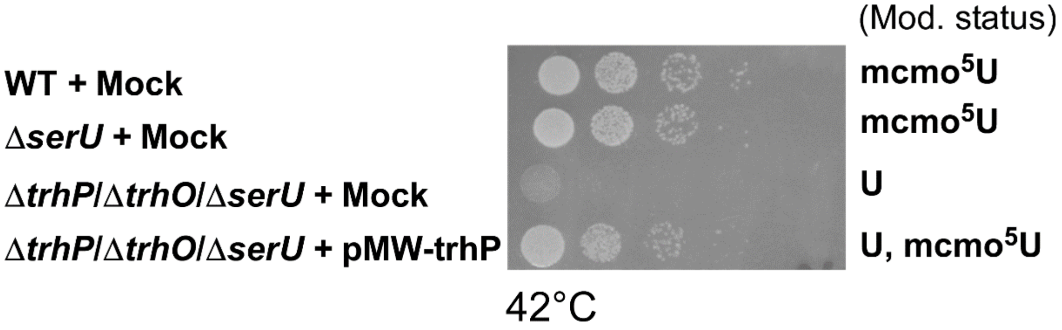

Supplementary Figure 8. Rescue of growth defect by introduction of plasmid-encoded *trhP*.

Growth properties of WT, Δ*serU*, Δ*trhP*/Δ*trhO*/Δ*serU* (mock plasmid), and Δ*trhP*/Δ*trhO*/Δ*serU* (pMW-trhP) strains. The wobble modification expected in each strain is shown on the right. Each strain was serially diluted (1:10), spotted onto LB agar plates containing 100 µg/ml ampicillin, and cultivated for 9 h at 42°C. Source data are provided as a Source Data File.

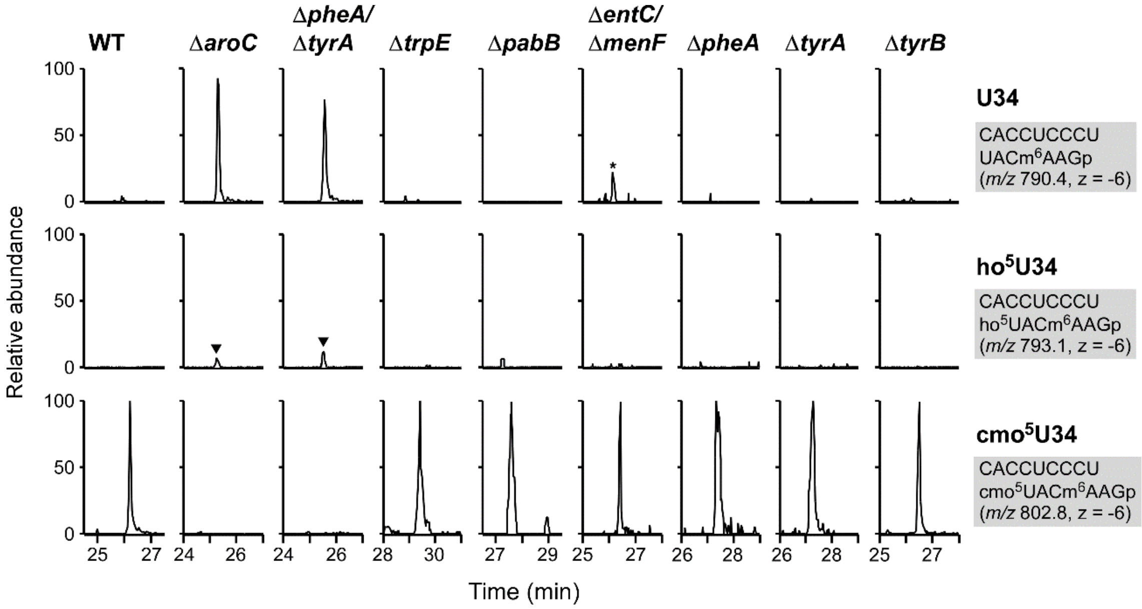

Supplementary Figure 9. Reverse genetic analysis of chorismate-related pathways in tRNA hydroxylation.

RNA-MS shotgun analysis of total RNA obtained from a series of *E. coli* knockout strains that lack genes involved in chorismate-related pathways (Figure 5A). XICs show the multiply charged negative ions of the anticodon-containing fragments of $tRNA^{Val1}$ with U34 (top panels), ho^5U34 (middle panels), and cmo^5U34 (bottom panels). The sequence, *m/z* value, and charge state of each fragment are shown on the right. Arrowheads indicate target peaks. The asterisk indicates a nonspecific peak with the same *m/z* value.

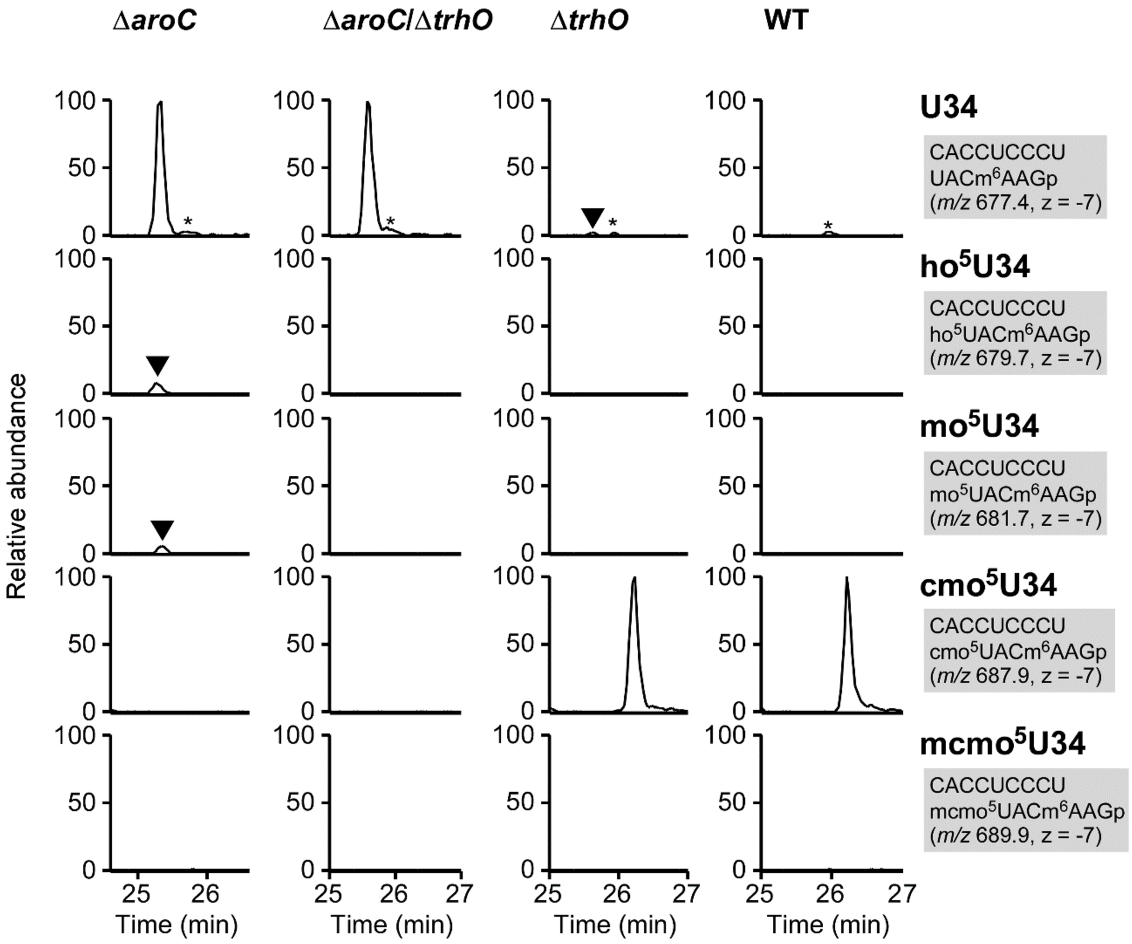

Supplementary Figure 10. Redundant formation of ho^{5}U34.

RNA-MS shotgun analyses of total tRNA obtained from *E. coli* Δ*aroC*, Δ*aroC*/Δ*trhO*, Δ*trhO*, and WT strains. XICs show the multiply charged negative ions of the anticodon-containing fragments of tRNA^{Val1} with U34 (top panels), ho^{5}U34 (second panels), mo^{5}U34 (third panels), cmo^{5}U34 (fourth panels), and mcmo^{5}U34 (bottom panels). Arrowheads indicate target peaks, and asterisks indicate unspecific peaks. The sequence, *m/z* value, and charge state of each fragment are shown on the right.

mo^{5}U34 observed in the Δ*aroC* strain is synthesized by CmoB, which methylates ho^{5}U using AdoMet instead of SCM-SAH [2-4].

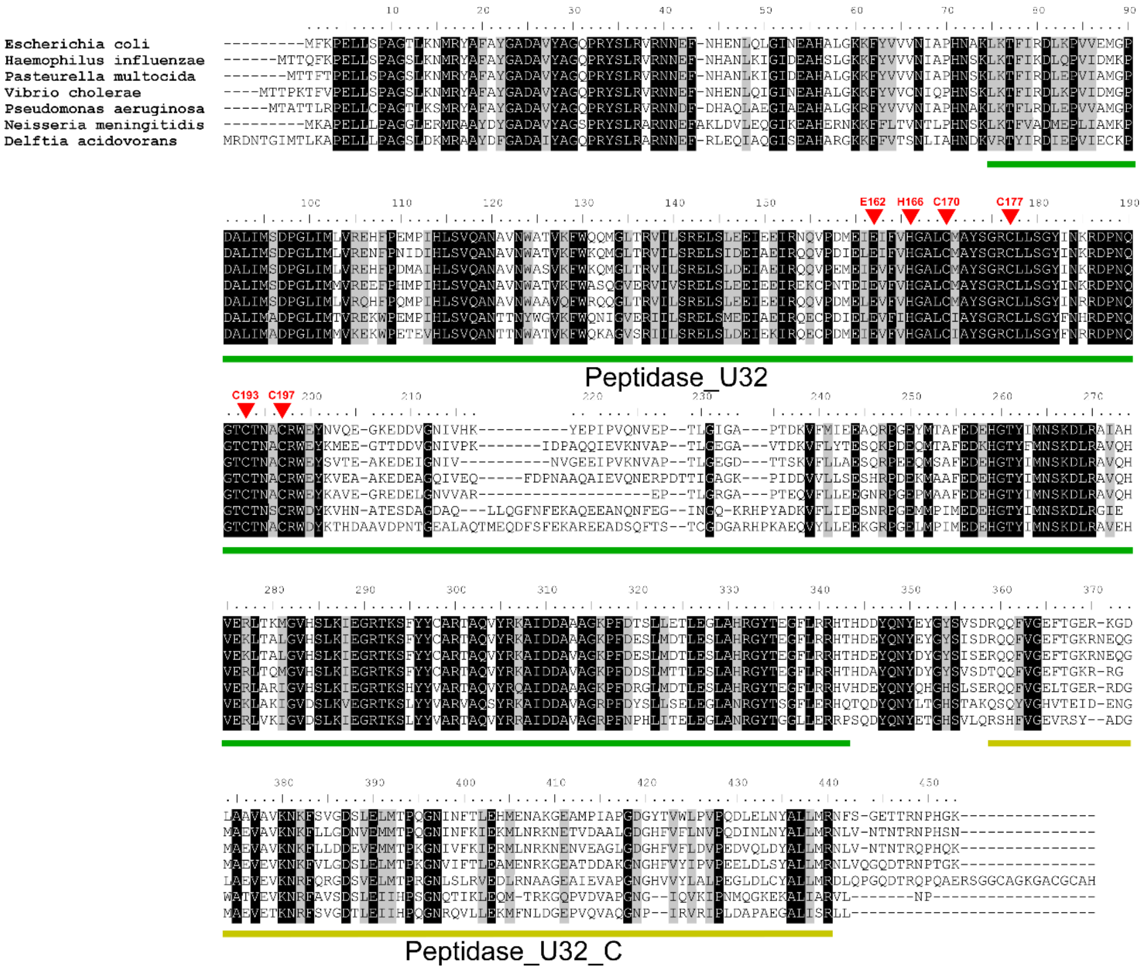

Supplementary Figure 11. Sequence alignment of TrhP.

Seven TrhP homologs of *Escherichia coli* (*yegQ*, NP_416585), *Haemophilus influenzae* (H0419, NP_438581), *Pasteurella multocida* (PM0233, AAK02317), *Pseudomonas aeruginosa* PAO1 (PA5440, NP_254127), *Vibrio cholerae* (VC0717, NP_230366), *Neisseria meningitidis* (NMB1664, NP_274669), and *Delftia acidovorans* (Daci_2456, ABX35094) were aligned by MAFFT. Identical and similar residues are shaded in black and gray, respectively. The two conserved motifs, Peptidase_U32 and Peptidase_U32_C, are indicated by green and yellow bars, respectively. Six essential residues of TrhP are indicated by red triangles.

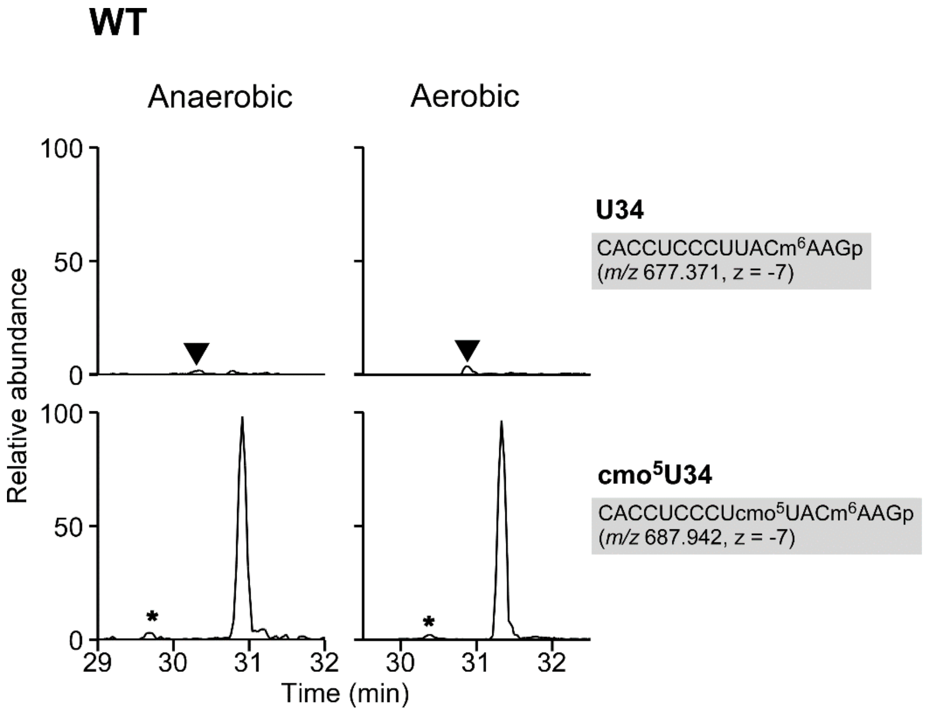

Supplementary Figure 12. Biogenesis of cmo^{5}U34 in the WT strain under aerobic and anaerobic conditions.

RNA-MS shotgun analyses of total tRNA obtained from the *E. coli* WT strain cultured under anaerobic (left panels) or aerobic (right panels) conditions. XICs show the multiply charged negative ions of the anticodon-containing fragments of $tRNA^{Val1}$ with U34 (upper panels) and cmo^{5}U34 (lower panels). Arrowheads indicate target peaks, and asterisks indicate unspecific peaks. The sequence, *m/z* value, and charge state of each fragment are shown on the right.

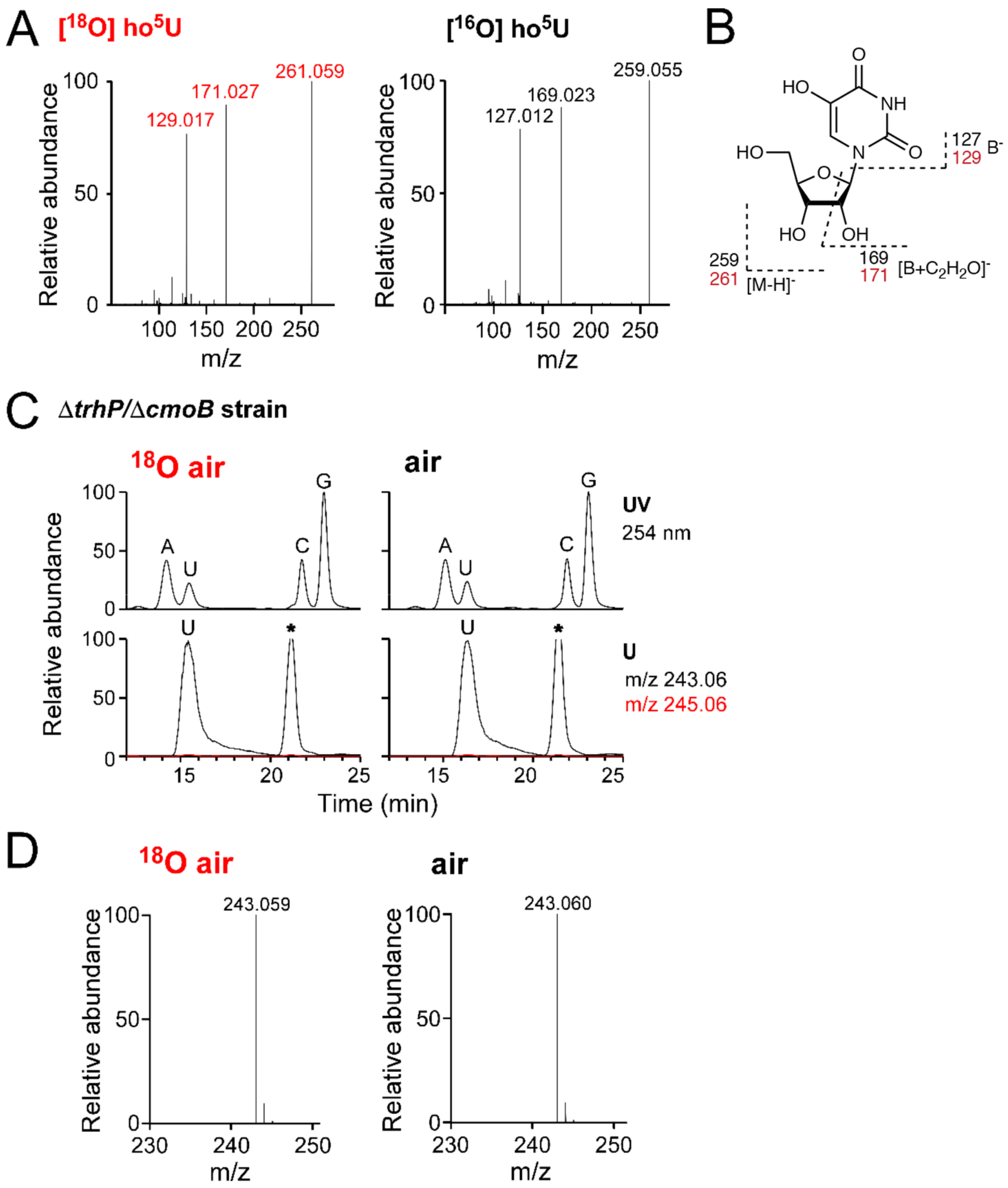

Supplementary Figure 13. Metabolic labeling of ho^{5}U with ^{18}O oxygen.

(A) CID spectra of ^{18}O-labeled (left panel) and non-labeled (right panel) ho^{5}U nucleosides observed in Figure 6B. (B) Product ions detected in the CID spectra [5], assigned on the chemical structure of the ho^{5}U nucleoside. (C) Nucleoside analyses of total RNAs obtained from the *E. coli* Δ*trhP*/Δ*cmoB* strain cultured in mixed gas with 20% $^{18}O_2$ (left panels) or in normal air (right panels). UV traces at 254 nm (upper panels) and XICs (lower panels) of deprotonated non-labeled (black line) and [^{18}O]-labeled (red line) U are shown. (D) Mass spectra of uridines from total RNAs analyzed in (C).

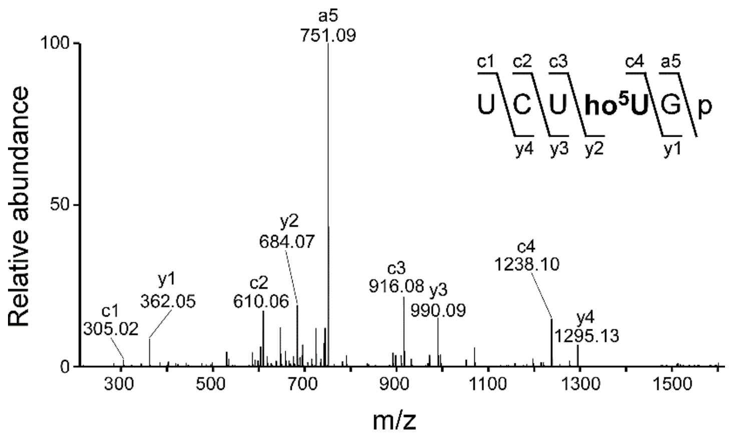

Supplementary Figure 14. TrhO-mediated *in vitro* reconstitution of ho^{5}U34, confirmed by CID.

A CID spectrum of the RNase T$_1$–digested fragment of *E. coli* tRNA^{Ser1} incubated with recombinant TrhO. The doubly charged negative ion of the ho^{5}U34-containing fragment (UCUho^{5}UGp, *m*/*z* 800.1, z = -2) was used as the precursor ion for CID. The product ions were assigned according to the literature [1]. The sequences of the parent ion and assigned product ions are described.

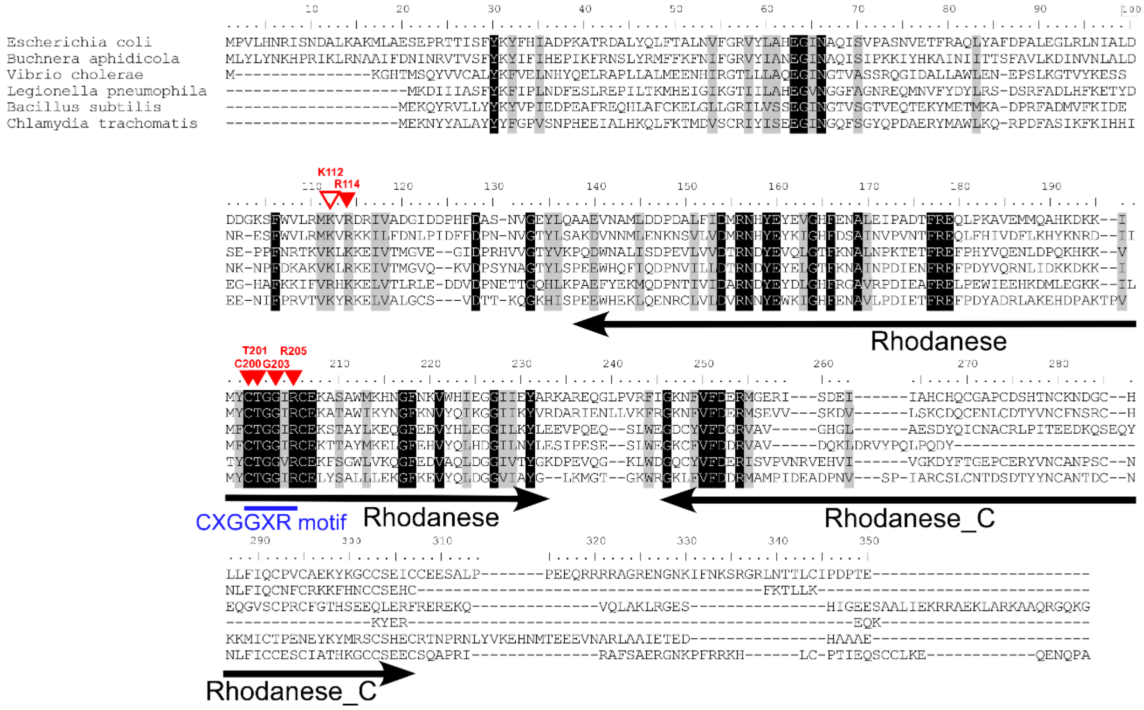

Supplementary Figure 15. Sequence alignment of TrhO homologs.

Six TrhO homologs of *Escherichia coli* (*yceA*, NP_415573), *Buchnera aphidicola* (*yceA*, AAO27051) *Vibrio cholerae* (VC1250, NP_230904), *Legionella pneumophila* (lpg2838, YP_096833), *Bacillus subtilis* (*ybfQ*, NP_388115), and *Chlamydia trachomatis* (*yceA*, NP_220144) were aligned by MAFFT. Identical and similar residues are shaded in black and gray, respectively. Filled red triangles indicate residues essential for genetic complementation, whereas open red triangles indicate residues that affect genetic complementation. Two motifs, Rhodanese and Rhodanase_C, are conserved within the subfamily. The conserved CXGGXR motif is indicated.

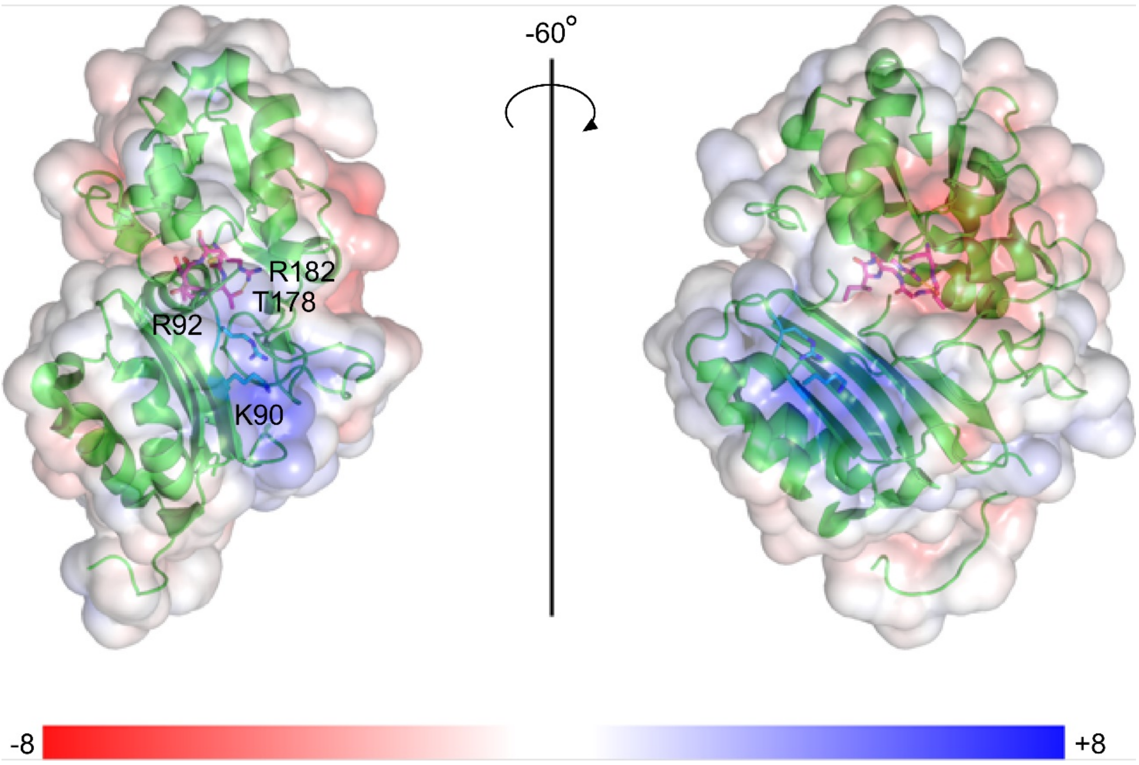

Supplementary Figure 16. Electrostatic potential of *L. pneumophila* TrhO homolog. The surface electrostatic potential of the Lpg2838 dimer (pdb: 4f67) was calculated by APBS [6] and overlaid on a green cartoon model generated by PyMol. Color codes indicate electrostatic potential; -8 kBT: red, +8 kBT: blue. Relative to the left structure, the right structure is rotated -60° around the z axis. The conserved CXGGXR motif is colored in magenta, and characteristic residues constituting the positively charged beta sheet are colored in cyan; both are displayed as stick models. K90, R92, T178, and R182 in Lpg2838 correspond to K112, R114, T201, and R205 in *E. coli* TrhO, respectively.

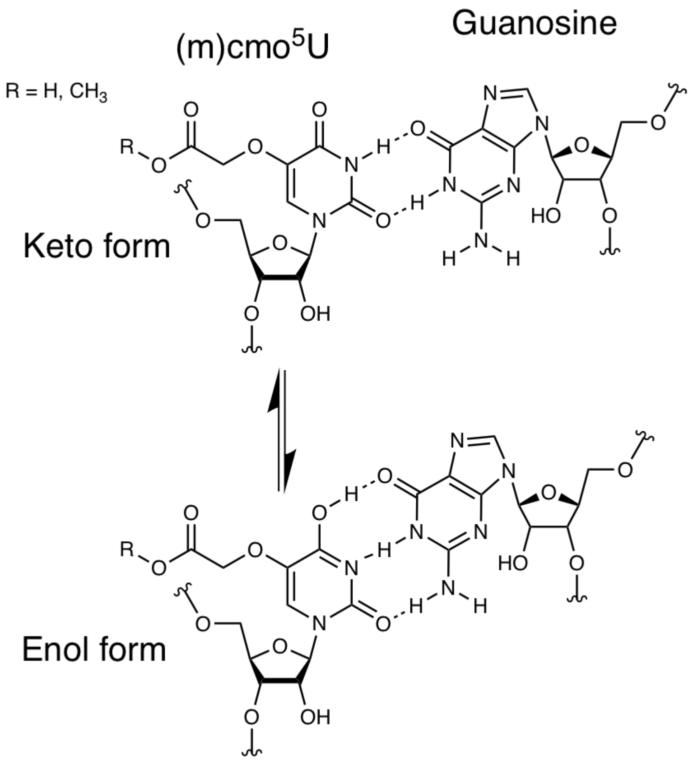

Supplementary Figure 17. Keto-enol tautomerisation of cmo^{5}U and basepairing with G.

During decoding, it is supposed that cmo^{5}U at wobble position adopts an enol form to base pair with guanosine in the Watson-Crick geometry.

Supplementary Table 1. List of *m/z* values of RNA fragments for use in calculation of modification frequencies.

tRNA	sequence	modification	m/z	
**$tRNA^{Ala1}$**	**CUUUGp**		z=-1	z=-2
(RNase T_1)		U34	1585.17	792.08
		ho^5U34	1601.16	800.08
		cmo^5U34	1659.17	829.08
		$mcmo^5U34$	1673.18	836.09
**$tRNA^{Leu3}$**	**AUUUAGp**		z=-2	z=-3
(RNase T_1)		U34	968.61	645.41
		ho^5U34	976.61	650.74
		cmo^5U34	1005.61	670.07
		$mcmo^5U34$	1012.62	674.75
**$tRNA^{Pro3}$**	**UmUUGp**		z=-1	z=-2
(RNase T_1)		U34	1294.14	646.57
		ho^5U34	1310.14	654.56
		cmo^5U34	1368.14	683.57
		$mcmo^5U34$	1382.16	690.57
**$tRNA^{Ser1}$**	**UCmUUGp**		z=-1	z=-2
(RNase T_1)		U34	1599.18	799.09
		ho^5U34	1615.18	807.09
		cmo^5U34	1673.18	836.09
		$mcmo^5U34$	1687.20	843.10
		$mcmo^5Um34$	1701.21	850.10
**$tRNA^{Thr4}$**	**ACmUUGp**		z=-1	z=-2
(RNase T_1)		U34	1622.21	810.60
		ho^5U34	1638.21	818.60
		cmo^5U34	1696.21	847.60
		$mcmo^5U34$	1710.23	854.61

tRNA^{Val1}	CACCUCCCUUACm^{6}AAGp	z=-3	z=-4	z=-5	z=-6	z=-7
(RNase T1)	U34	1581.88	1186.15	948.72	790.43	677.37
	ho^{5}U34	1587.21	1190.15	951.92	793.10	679.66
	cmo^{5}U34	1606.54	1204.65	963.52	802.77	687.94
	mcmo^{5}U34	1611.21	1208.16	966.33	805.10	689.95

Supplementary Table 2. List of strains used in this study.

***E. coli* strains**	**Source**
**BW25113**	NBRP (Parent strain)
**BW25113Δ*yegQ*::KanR**	NBRP
**BW25113Δ*ydcP*::KanR**	NBRP
**BW25113 Δ*yhbU-yhbV*::CmR**	This study
**BW25113 Δ*yegQ*::KanR Δ*yhbU-yhbV*::CmR**	This study
**BW25113 Δ*yegQ*<>frt Δ*yhbU-yhbV*::CmR**	This study
**BW25113 Δ*yegQ*<>frt Δ*ydcP*::KanR Δ*yhbU-yhbV*::CmR**	This study
**BW25113 Δ*yceA*::KanR**	NBRP
**BW25113 Δ*yegQ*<>frt**	This study
**BW25113 Δ*yegQ*<>frt Δ*yceA*::KanR**	This study
**BW25113 Δ*smtA*::KanR**	NBRP
**BW25113 Δ*cmoB*::KanR**	NBRP
**BW25113 Δ*serU*::CmR**	This study
**BW25113 Δ*smtA*::KanR Δ*serU*::CmR**	This study
**BW25113 Δ*cmoB*::KanR Δ*serU*::CmR**	This study
**BW25113 Δ*yegQ*<>frt Δ*yceA*::KanR Δ*serU*::CmR**	This study
**BW25113 Δ*yegQ*::KanR Δ*serU*::CmR**	This study
**BW25113 Δ*yceA*::KanR Δ*serU*::CmR**	This study
**BW25113 Δ*thrW*::CmR**	This study
**BW25113 Δ*smtA*::KanR Δ*thrW*::CmR**	This study
**BW25113 Δ*cmoB*::KanR Δ*thrW*::CmR**	This study
**BW25113 Δ*yegQ*<>frt Δ*yceA*::KanR Δ*thrW*::CmR**	This study
**BW25113 Δ*yegQ*::KanR Δ*thrW*::CmR**	This study
**BW25113 Δ*yceA*::KanR Δ*thrW*::CmR**	This study
**BW25113 Δ*proK*::KanR**	This study
**BW25113 Δ*smtA*<>frt Δ*proK*::KanR**	This study
**BW25113 Δ*cmoB*<>frt Δ*proK*::KanR**	This study
**BW25113 Δ*yegQ*<>frt Δ*yceA*<>frt Δ*proK*::KanR**	This study
**BW25113 Δ*aroC*::KanR**	NBRP
**BW25113 Δ*yceA*<>frt**	This study
**BW25113 Δ*yceA*<>frt Δ*aroC*::KanR**	This study
**BW25113 Δ*trpE*::KanR**	NBRP
**BW25113 Δ*pabB*::KanR**	NBRP
**BW25113 Δ*pheA*::KanR**	NBRP

**BW25113 Δ*tyrA*::KanR**	NBRP
**BW25113 Δ*tyrB*::KanR**	NBRP
**BW25113 Δ*pheA-tyrA*::CmR**	This study
**BW25113 Δ*entC*::KanR**	NBRP
**BW25113 Δ*menF*::KanR**	NBRP
**BW25113 Δ*entC*<>frt**	This study
**BW25113 Δ*entC*<>frt Δ*menF*::KanR**	This study
**BW25113 Δ*yceA*::KanR Δ*pheA-tyrA*::CmR**	This study
**BW25113 Δ*yceA*<>frt Δ*pheA-tyrA*::CmR**	This study
**BW25113 Δ*cmoA*::KanR**	NBRP
**BW25113 Δ*yceA*<>frt Δ*pheA-tyrA*::CmR Δ*cmoA*::KanR**	This study
**BW25113 Δ*cmoB*<>frt**	NBRP
**BW25113 Δ*cmoB*<>frt Δ*yegQ*::KanR**	This study

***B. subtilis* strains**	**Source**
**168 trpC2 (WT)**	NBRP (parent strain)
**Δ*yrrO* (*trhP1*)**	NBRP
**Δ*yrrN* (*trhP2*)**	NBRP
**Δ*ybfQ* (*trhO*)**	NBRP
**Δ*yrrM* (*trmR*)**	This study
**Δ*yrrN* Δ*yrrO***	This study
**Δ*yrrN* Δ*yrrO* Δ*ybfQ***	This study

Supplementary References

1 McLuckey, S. A., Van Berkel, G. J. & Glish, G. L. Tandem mass spectrometry of small, multiply charged oligonucleotides. *Journal of the American Society for Mass Spectrometry* **3**, 60-70, doi:10.1016/1044-0305(92)85019-G (1992).

2 Nasvall, S. J., Chen, P. & Bjork, G. R. The modified wobble nucleoside uridine-5-oxyacetic acid in tRNAPro(cmo5UGG) promotes reading of all four proline codons in vivo. *RNA* **10**, 1662-1673 (2004).

3 Kim, J. *et al.* Structure-guided discovery of the metabolite carboxy-SAM that modulates tRNA function. *Nature* **498**, 123-126, doi:10.1038/nature12180 (2013).

4 Hagervall, T. G., Jönsson, Y. H., Edmonds, C. G., McCloskey, J. A. & Bjork, G. R. Chorismic acid, a key metabolite in modification of tRNA. *J. Bacteriol.* **172**, 252-259 (1990).

5 Liu, J. *et al.* A theoretical and mass spectrometry study of the novel mechanism of N-glycosidic bond cleavage in nucleoside. *International Journal of Mass Spectrometry* **282**, 1-5, doi:10.1016/j.ijms.2009.01.003 (2009).

6 Baker, N. A., Sept, D., Joseph, S., Holst, M. J. & McCammon, J. A. Electrostatics of nanosystems: Application to microtubules and the ribosome. *Proceedings of the National Academy of Sciences of the United States of America* **98**, 10037-10041, doi:10.1073/pnas.181342398 (2001).
