# Supplementary material for: Dual pathways of tRNA hydroxylation ensure efficient translation by expanding decoding capability
Source: Nat Commun. 2019 Jun 28;10:2858. doi: 10.1038/s41467-019-10750-8 (PMC6599085; doi:10.1038/s41467-019-10750-8)
Supplement: Supplementary file 4 — Description of Additional Supplementary Files [file 41467_2019_10750_MOESM4_ESM.docx]

**Description of Additional Supplementary Files**

File Name: Supplementary Data 1.
Description: List of peptidase U32 motifs deposited in Pfam. 3,521 proteins bearing peptidase U32 motif are listed with organism names and subfamilies of the motif. Supplementary Figure 1 is generated by this data set.

File Name: Supplementary Data 2.
Description: Phylogenetic distribution of the genes responsible for xo5U synthesis A series of genes responsible for xo5 U biogenesis are compiled for organisms registered in Pfam. Supplementary Figure 6 is generated by this data set.

File Name: Supplementary Data 3.
Description: List of primers used in this study.
