# Supplementary material for: Dual pathways of tRNA hydroxylation ensure efficient translation by expanding decoding capability
Source: Nat Commun. 2019 Jun 28;10:2858. doi: 10.1038/s41467-019-10750-8 (PMC6599085; doi:10.1038/s41467-019-10750-8)
Supplement: Supplementary file 2 — Peer Review Files [file 41467_2019_10750_MOESM2_ESM.pdf]

Reviewers' comments:

Reviewer #1 (Remarks to the Author):

Dual pathways of tRNA hydroxylation.....
Sakai, Kimura, and Suzuki

In this manuscript the two pathways that lead to the formation of ho(5)U in bacteria are elucidated, their respective mechanisms and substrate specificities are determined, and the role of the different moieties in the cmo(5)U modification in codon recognition are dissected.

This is truly a fantastic paper, both because of its technical excellence and for its scientific significance. It completes our understanding of the pathways that lead to the formation of xmo(5)U in anticodons, and it opens a fascinating set of questions regarding the physiological relevance and the evolution of this modified bases.

The most astonishing result to me is the discovery of the partial functional redundancy of TrhP and TrhO. The authors show that TrhP is a general modifier of tRNAs for TSAVPL, while TrmO complements this activity under aerobic conditions to modify only TSAV tRNAs. Clearly, O2 exposure may be the main factor that determines which enzymes are present where, but many additional questions emerge from this, such as how the difference in enzymes influences the proteomes of the species that contain them.

I only have major reservations regarding the evolutionary analysis presented in the results section 'Phylogenetic distribution of trhP and trhO'. It is very clear from the phylogenetic tree presented in figure S1 that TrhP and ThrP1 are related enzymes, while TrhP2 is a less related structure. Possibly TrhP2 dimerized with TRhP to generate a heterodimeric TrhP1/TrhP2 enzyme that has two active sites and loses activity when one subunit is missing. I do not know if that's true, but the conclusion that is put forward: 'the mutually exclusive distribution of ThrP and ThrP1/ThrP2 suggests that these two types of proteins evolved independently' is certainly denied by figure S1.

In the same section, and from the percentages of distribution of the genes, the authors conclude that the coexistence of ThrP (or ThrP1/ThrP2) with ThrO provides a fitness advantage. I don't think this conclusion is warranted. The two enzymes may coexist because they are required under different growth conditions, and they are not necessarily providing a fitness advantage in front of other organisms in each individual growth condition.

Minor points:
Reference 26 should be changed to the specific reference for the fact cited.
Page 7, line 7. replace 'these knock-out strains' by 'the respective knock-out strains'.
Page 8, line 12. 'mo5U is replaced by'
Page 12, line 7. 'their ability to be decoded by'

Reviewer #2 (Remarks to the Author):

This work describes the discovery of two E. coli genes that code for enzymes that catalyze the ho5U34 modification in several wobble positions of tRNA, and the characterization of these gene functions in E. coli deletion strains. Formation of ho5U34 is the first step of a cascade of enzyme reactions that eventually generate the cmo5U34 (and its methylated form mcmo5U34) in these tRNAs. The two enzymes described in this work catalyze this reaction in redundant pathways; they are the last unknown in this reaction cascade. This work is a tour-de-force starting from phylogenetic analysis for candidate genes and goes all the way to identify the reactants used (including molecule oxygen), and

the growth phenotypes of several deletion mutants in different E. coli genetic backgrounds. The LC/MS data are of high quality and unambiguous; there are also complementary experiments performed to prove that these are the right enzymes for this reaction.

Major comment:
My main comments deal with the decoding experiments (Fig. 4D, 4E). Fig. 1B suggests that the main function of cmo5U34/mcmo5U34 seems to be expanding the decoding capacity of several U34-tRNAs. Yet, this idea was not tested. Instead, Fig. 4 experiments used a frameshift reporter, and the goal seemed to be testing that cmo5U34 modified tRNAs prefer G-ending over A-ending codon.

1. If demonstrating decoding preference for cmo5U34 modified tRNA is the purpose, constructs containing GCA codon for GCG, and UCA codon for UCG construct need to be tested to make this point.

2. The reporter construct used here requires decoding in the context of a frameshift sequence. This is unusual as common tests for decoding efficiency is to compare the protein levels using a run of codons such as (GCG)6 and (GCA)6 between the two luciferase genes. It is unclear why this straightforward test was not done.

3. The reason for the observed phenotypes may not even be preferred decoding of G-ending codons. Rather, the author's introduction refers to the possibility of expanding the decoding capability (Fig. 1B) as a potential main function for the cmo5U34 modification. This idea should be tested but was not. The authors could test the decoding efficiency of U-ending codons in their deletion strains.

Minor comment:
1. It is unclear how many tRNAs are substrates for the TrhO enzyme. From Fig. 2C it looks like 4 of the 6 tRNAs are TrhO substrates, is this correct? This information should be included in the summary Fig. 7.

Reviewer #3 (Remarks to the Author):

In the work presented by Sakai, Kimura and Suzuki the authors analyze the biological hydroxylation of uridine commonly found at position 34 of tRNAs in bacteria. In bacteria, all post-transcriptional tRNA modifying are known, except the enzyme for acp3U and ho5U. With this work, the authors describe their search and subsequent success in finding the enzyme(s) responsible for ho5U. In fact, 2 enzymes are identified, TrhP and TrhO, which are responsible for the hydroxylation reaction. Furthermore, the authors describe the metabolites involved in ho5U formation. Intriguingly, for trhO, molecular oxygen is the oxygen source of the hydroxyl-group. In addition, to these detailed studies on the enzymatic functions of TrhP and TrhO, the authors were able to describe the impact of lost ho5U formation on bacterial growth. Here, they describe a growth phenotype in E. coli if both TrhP and TrhO are absent. The authors thus provide a) a detailed pathway on the formation of hypermodified uridine derivatives in bacteria and b) functional insight into the importance of the uridine 5-hydroxylation. The manuscript is very well crafted and is easy to read. All data is shown in logical order and the conclusions are emphasized by the presented data. The presented findings are of high importance and significance to the field. Nevertheless, this reviewer has a few points that should be addressed before acceptance.

1) Figure 2C: The displayed modification density is from mass spectrometric analysis of T1 digests. The areas of differently modified and unmodified fragments do not necessarily reflect the abundance of the fragments and thus a relative quantification is not possible without calibration or internal standards. The ionization of some fragments might be more efficient and thus they appear over-represented. How did the authors verify the quantitative nature of their measurements?

2) the section “Phenotypic features of E. coli strain lacking tRNA hydroxylation” is potentially difficult to understand for non-tRNA experts. The authors should consider referencing Figure 1B more generous to facilitate a better understanding.

3) The impact of decoding is only shown for tRNASer2 and tRNAAla but not the other ho5U-modified tRNAs. Although only 2 tRNAs responsible for G-ending codons are analyzed, the authors extrapolate their observation to all tRNAs for G-ending codons. Either the authors present evidence that their statement is true for all respective tRNAs or they soften their statement to only include the two analyzed tRNAs.

4) In the TrhO hydroxylation section with oxygen-18, why do the authors not show data for 16O- and 18O-uridine? With their MS setup, this is a simple measurement which clearly shows that the 18O must be at position 5. Alternatively, a MS3 spectrum of ho5U would clearly validate the position.
5) The discussion is jumping from TrhP to TrhO to TrhP and TrhO again. The authors should reconsider re-arrangement of the discussion for better readability.

Reviewer #4 (Remarks to the Author):

The manuscript entitled „Dual pathways of tRNA hydroxylation ensure efficient translation by expanding decoding capability‟ provides a very comprehensive and detailed insight into the formation and role of U34 modifications within various tRNAs in E. coli and also B. subtilis. This study revealed essential enzymes involved in the biogenesis of mcmo5U34 and derivatives thereof and also provides some first insights into essential domains of these enyzmes. A large amount of experiments and assays were performed to substantiate their findings and conclusions. I only have a few small comments for the authors to be addressed.

In the introduction (page 3) the authors state U34 can base pair with A and G. This somehow indicates that only U34 can do this but to my knowledge also G34 can provide wobble base pairing.

On page 4 (at the top) the authors state that they find cmoU34 in a variety of tRNAs. For clarity I would suggest to include that this was found in E. coli (in the sentence before B. subtilis was mentioned).

To make this complex topic easier maybe a supplemental figure could help to understand the role of cmo5U during base pairing at the wobble position and how and why it determines the respective geometry or tautomeric features.

In Figure 2E, I would recommend to insert tRNAVal into the figure to clarify which tRNA was analyzed (in analogy to Fig. 2A and 2B).

First of all, we appreciate all reviewers for spending your precious time to review our manuscript, and giving us a number of productive comments to improve it. Revised descriptions in the main text are marked in yellow.

Response to Reviewer #1's comments

Dual pathways of tRNA hydroxylation.....
Sakai, Kimura, and Suzuki

In this manuscript the two pathways that lead to the formation of ho(5)U in bacteria are elucidated, their respective mechanisms and substrate specificities are determined, and the role of the different moieties in the cmo(5)U modification in codon recognition are dissected.

This is truly a fantastic paper, both because of its technical excellence and for its scientific significance. It completes our understanding of the pathways that lead to the formation of xmo(5)U in anticodons, and it opens a fascinating set of questions regarding the physiological relevance and the evolution of this modified bases.

Thank you very much for deep understanding of our work and positive comments.

The most astonishing result to me is the discovery of the partial functional redundancy of TrhP and TrhO. The authors show that TrhP is a general modifier of tRNAs for TSAVPL, while TrmO complements this activity under aerobic conditions to modify only TSAV tRNAs. Clearly, O2 exposure may be the main factor that determines which enzymes are present where, but many additional questions emerge from this, such as how the difference in enzymes influences the proteomes of the species that contain them.

Thank you for this insightful remark. Yes, we need to consider how these two pathways influence entire proteome through regulatory decoding mediated by the different sets of tRNAs.

I only have major reservations regarding the evolutionary analysis presented in the results section 'Phylogenetic distribution of trhP and trhO'. It is very clear from the phylogenetic tree presented in figure S1 that TrhP and ThrP1 are related enzymes, while TrhP2 is a less related structure. Possibly TrhP2 dimerized with TRhP to generate a heterodimeric TrhP1/TrhP2 enzyme that has two active sites and loses activity when one subunit is missing. I do not know if that's true, but the conclusion that is put forward: 'the mutually exclusive distribution of ThrP and ThrP1/ThrP2 suggests that these two types of proteins evolved independently' is

certainly denied by figure S1.

As pointed out, we realized our irrelevant description on the evolutional aspect of the two systems. In fact, TrhP1 is branched out from TrhP, then evolved to require TrhP2. Thus, we rephrased this part as follows.
“Given that TrhP1 is a related family with TrhP (Figure S1), TrhP1 might have branched out from TrhP, and evolved to require a paralogous protein TrhP2 that might be generated by gene duplication. Supporting this speculation, trhP1 and trhP2 are tandemly encoded in the same operon in *B. subtilis* (Figure 2D).”

In the same section, and from the percentages of distribution of the genes, the authors conclude that the coexistence of ThrP (or ThrP1/ThrP2) with ThrO provides a fitness advantage. I don't think this conclusion is warranted. The two enzymes may coexist because they are required under different growth conditions, and they are not necessarily providing a fitness advantage in front of other organisms in each individual growth condition.

As suggested, we rephrased this part as follows.
“This significant overlap suggests that harboring both pathways for tRNA hydroxylation might help organisms to adapt to two different environments, i.e., aerobic and anaerobic conditions.”

Minor points:
Reference 26 should be changed to the specific reference for the fact cited.

We put appropriate references here.

Page 7, line 7. replace 'these knock-out strains' by 'the respective knock-out strains'.

It was rephrased as suggested.

Page 8, line 12. 'mo5U is replaced by'

It was corrected.

Page 12, line 7. 'their ability to be decoded by'

It was corrected.

Reviewer #2 (Remarks to the Author):

This work describes the discovery of two E. coli genes that code for enzymes that catalyze the ho5U34 modification in several wobble positions of tRNA, and the characterization of these gene functions in E. coli deletion strains. Formation of ho5U34 is the first step of a cascade of enzyme reactions that eventually generate the cmo5U34 (and its methylated form mcmo5U34) in these tRNAs. The two enzymes described in this work catalyze this reaction in

redundant pathways; they are the last unknown in this reaction cascade. This work is a tour-de-force starting from phylogenetic analysis for candidate genes and goes all the way to identify the reactants used (including molecule oxygen), and the growth phenotypes of several deletion mutants in different E. coli genetic backgrounds. The LC/MS data are of high quality and unambiguous; there are also complementary experiments performed to prove that these are the right enzymes for this reaction.

We appreciate deep understanding of our work, positive comments and a lot of encouragement.

Major comment:
My main comments deal with the decoding experiments (Fig. 4D, 4E). Fig. 1B suggests that the main function of cmo5U34/mcmo5U34 seems to be expanding the decoding capacity of several U34-tRNAs. Yet, this idea was not tested. Instead, Fig. 4 experiments used a frameshift reporter, and the goal seemed to be testing that cmo5U34 modified tRNAs prefer G-ending over A-ending codon.

To examine decoding ability of ho^5U34 toward Y-ending codons, we need to disrupt isoacceptor tRNAs responsible for Y-ending codons. According to the *E. coli* decoding rule (Figure 1B), Ala2, Val2 and Pro2 can be deleted, because respective Y-ending codons are redundantly decoded by tRNAs bearing (m)cmo^5U34. Thus, we tried to disrupt each of these tRNA genes in Δ*trhP*/Δ*trhO* strain. Only Pro2 (*proL*) could be deleted to give Δ*trhP*/Δ*trhO*/Δ*proL* strain, due to a technical difficulty of knocking out the other two tRNAs. However, we did not observe any significant synthetic phenotypes by the knockout of Pro2 gene with the deletion of *trhP* and *trhO* (Figure A), suggesting that $mcmo^5U34$ modification does not contribute to the decoding of CCU/CCC codons, even in the absence of $tRNA^{Pro2}$ with GGG anticodon. So, we did not test these Y-ending codons using our reporter assay system. In contrast, as we showed in Figure 4, we detected clear phenotypes in the strains in which tRNAs bearing (m)cmo^5U is a sole isoacceptor to decode G-ending codons. Therefore, in this paper, we focus on decoding ability of ho^5U34 toward G-ending codon.

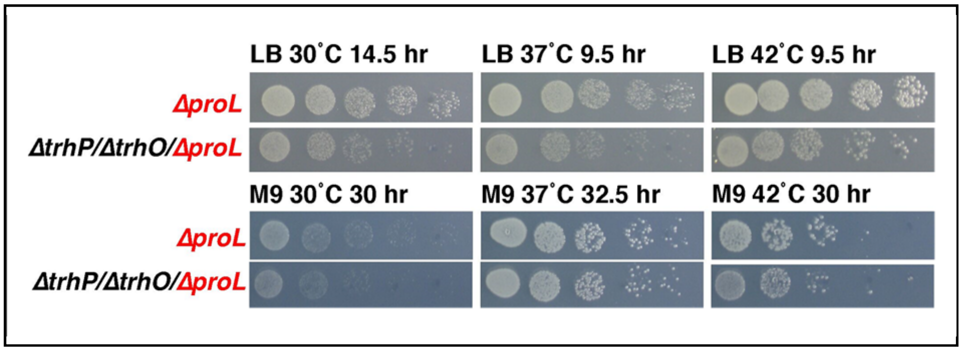

Figure A. Growth properties of Δ*proL*, and Δ*trhP*/Δ*trhO*/Δ*proL* strains.

1. If demonstrating decoding preference for cmo5U34 modified tRNA is the purpose, constructs containing GCA codon for GCG, and UCA codon for UCG construct need to be tested to make this point.

The reporter construct based on RF2-recoding mechanism does not aim to compare decoding efficiency between A-ending codon and G-ending codon. In this construct, decoding ability of tRNAs with various modifications can be measured by competition with the +1 frameshifting. This system is well-established, and has been applied to measure decoding ability of xo^5U-type modifications.

As this reviewer suggested, a reporter construct with a run of test codons ie $(GCG)_6$ and $(GCA)_6$ between the two luciferases might work for this purpose. However, we don't know how sensitive of the construct, and nobody examined this kind of construct for cmo^5U modifications so far.

2. The reporter construct used here requires decoding in the context of a frameshift sequence. This is unusual as common tests for decoding efficiency is to compare the protein levels using a run of codons such as (GCG)6 and (GCA)6 between the two luciferase genes. It is unclear why this straightforward test was not done.

The reporter construct we used here was originally developed by Curran and Yarus (JMB, 1989). Glenn Bjork's group elegantly adopted this system to examine decoding ability of cmo^5U modification in several mutant strains (Nasvall et al., 2007). Our previous CmoM paper used this construct, because it was sensitive enough to detect small difference in the decoding activity with and without terminal methyl modification (Sakai et al., 2016). Thus, this reporter system has been well-established especially for testing $(m)cmo^5U$ modifications. In fact, we clearly showed different decoding ability between U34 and ho^5U34 for UCG codon in the absence of $tRNA^{Ser2}$.

3. The reason for the observed phenotypes may not even be preferred decoding of G-ending codons. Rather, the author's introduction refers to the possibility of expanding the decoding capability (Fig. 1B) as a potential main function for the cmo5U34 modification. This idea should be tested but was not. The authors could test the decoding efficiency of U-ending codons in their deletion strains.

As discussed earlier, to examine the decoding efficiency of Y-ending codons in our deletion strains, we need to further disrupt isoacceptor tRNAs responsible for Y-ending codons, such as Ala2, Val2 and Pro2. As described above, we only disrupted Pro2 (*proL*) to obtain Δ*trhP*/Δ*trhO*/Δ*proL* strain. However, we did not observe any significant phenotype of this strain when compared to Δ*proL* strain (Figure A in our response to Reviewer 2). This is the reason why we did not test the Y-ending codons in our constructs. However, as pointed out by this reviewer, we need to test decoding ability of (m)cmo5U modification for Y-ending codons in our future study. To this end, we should establish more sensitive constructs or methods to measure the decoding abilities of tRNAs with various modifications.

Minor comment:

1. It is unclear how many tRNAs are substrates for the TrhO enzyme. From Fig. 2C it looks like 4 of the 6 tRNAs are TrhO substrates, is this correct? This information should be included in the summary Fig. 7.

As suggested, we put this information in Figure 7.

Reviewer #3 (Remarks to the Author):

In the work presented by Sakai, Kimura and Suzuki the authors analyze the biological hydroxylation of uridine commonly found at position 34 of tRNAs in bacteria. In bacteria, all post-transcriptional tRNA modifying are known, except the enzyme for acp3U and ho5U. With this work, the authors describe their search and subsequent success in finding the enzyme(s) responsible for ho5U. In fact, 2 enzymes are identified, TrhP and TrhO, which are responsible for the hydroxylation reaction. Furthermore, the authors describe the metabolites involved in ho5U formation. Intriguingly, for trhO, molecular oxygen is the oxygen source of the hydroxyl-group. In addition, to these detailed studies on the enzymatic functions of TrhP and TrhO, the authors were able to describe the impact of lost ho5U formation on bacterial growth. Here, they describe a growth phenotype in E. coli if both TrhP and TrhO are absent. The authors thus provide a) a detailed pathway on the formation of hypermodified uridine derivatives in bacteria and b) functional insight into the importance of the uridine 5-hydroxylation.
The manuscript is very well crafted and is easy to read. All data is shown in logical order and the conclusions are emphasized by the presented data. The presented findings are of high importance and significance to the field. Nevertheless, this reviewer has a few points that should be addressed before acceptance.

Thank you very much for deep understanding and positive comments.

1) Figure 2C: The displayed modification density is from mass spectrometric analysis of T1 digests. The areas of differently modified and unmodified fragments do not necessarily reflect the abundance of the fragments and thus a relative quantification is not possible without calibration or internal standards. The ionization of some fragments might be more efficient and thus they appear over-represented. How did the authors verify the quantitative nature of their measurements?

Basically, in negative mode of ESI, the ionization efficiencies of RNA fragments bearing the same sequence but different modification do not differ largely, because ESI ionization relies mainly on the number of phosphate groups rather than the type of base modifications (Ohira et al., 2016). In fact, we previously measured ESI detection efficiency of RNase T1 fragment with or without t^6A modification, and confirmed the same ionization efficiency (Lin et al., *Nature Commun*, 2018). Thus, using the peak intensity ratio of RNase T1 fragments with $mcmo^5Um$, $mcmo^5U$, cmo^5U, and U, we relatively quantify the frequency of each modification. We put this information in the text. However, for precise quantification, we need to have an authentic oligo for each modified fragment for absolute quantification.

2) the section “Phenotypic features of E. coli strain lacking tRNA hydroxylation” is potentially difficult to understand for non-tRNA experts. The authors should consider referencing Figure 1B more generous to facilitate a better understanding.

In the legend for Figure 1B, we explained a general decoding rule with isoaccpeptors for non-tRNA experts.

3) The impact of decoding is only shown for tRNASer2 and tRNAAla but not the other ho5U-modified tRNAs. Although only 2 tRNAs responsible for G-ending codons are analyzed, the authors extrapolate their observation to all tRNAs for G-ending codons. Either the authors present evidence that their statement is true for all respective tRNAs or they soften their statement to only include the two analyzed tRNAs.

To analyze the impact on the decoding for G-ending codons, we need to disrupt tRNAs responsible for G-ending codons. According to the *E. coli* decoding rule (Figure 1B), tRNAs for Leu1, Pro1, Ser2, and Thr2 are responsible for G-ending codons. We could not disrupt Leu genes, because there are 4 copies, whereas each of the other 3 tRNA genes was disrupted, and we found clear genetic interaction with *trhP* and *trhO* (Figure 4A). Thus, we can stress the importance of ho^5U modification in decoding of CCG, UCG, and ACG. However, regarding the reporter assay, we only tested GCG and UCG codons. Thus, as suggested by this reviewer, we softened our statement, and mentioned only GCG and UCG codons that we examined in this study.

4) In the TrhO hydroxylation section with oxygen-18, why do the authors not show data for 16O- and 18O-uridine? With their MS setup, this is a simple measurement which clearly shows that the 18O must be at position 5. Alternatively, a MS3 spectrum of ho5U would clearly validate the position.

As requested, we added data for mass chromatograms (Figure S13C) and spectra (Figure S13D) for uridines in both ^{18}O and normal air conditions. Little ^{18}O in uridine was detected.

5) The discussion is jumping from TrhP to TrhO to TrhP and TrhO again. The authors should reconsider re-arrangement of the discussion for better readability.

Thank you very much for this helpful advice. We reorganize the paragraphs in discussion accordingly.

Reviewer #4 (Remarks to the Author):

The manuscript entitled „Dual pathways of tRNA hydroxylation ensure efficient translation by expanding decoding capability“ provides a very comprehensive and detailed insight into the formation and role of U34 modifications within various tRNAs in E. coli and also B. subtilis. This study revealed essential enzymes involved in the biogenesis of mcmo5U34 and derivatives thereof and also provides some first insights into essential domains of these enyzmes. A large amount of experiments and assays were performed to substantiate their findings and conclusions.

We appreciate these positive comments.

I only have a few small comments for the authors to be addressed.

In the introduction (page 3) the authors state U34 can base pair with A and G. This somehow indicates that only U34 can do this but to my knowledge also G34 can provide wobble base pairing.

As suggested, we put information that G34 pairs with U and C in the introduction.

On page 4 (at the top) the authors state that they find cmoU34 in a variety of tRNAs. For clarity I would suggest to include that this was found in E. coli (in the sentence before B. subtilis was mentioned).

As suggested, we included that it was found in *E. coli*.

To make this complex topic easier maybe a supplemental figure could help to understand the role of cmo5U during base pairing at the wobble position and how and why it determines the respective geometry or tautomeric features.

We appreciate this practical suggestion. We made a supplemental figure (Figure S17) to describe the base pairing geometry of tautomeric forms.

In Figure 2E, I would recommend to insert tRNAVal into the figure to clarify which tRNA was analyzed (in analogy to Fig. 2A and 2B).

As suggested, we put this information.

REVIEWERS' COMMENTS:

Reviewer #1 (Remarks to the Author):

All my concerns have been satisfactorily addressed. Thank you.

Reviewer #2 (Remarks to the Author):

The authors adequately addressed my comments.

Reviewer #3 (Remarks to the Author):

All my remarks were addressed. This is a great manuscript which should be published asap.

Reviewer #4 (Remarks to the Author):

All points raised were addressed appropriately by the authors. It is a really exciting mansucript!
